# Oral Microbial Extracellular Vesicles as Novel Mediators of Alzheimer’s Pathogenesis: A Critical Review of the Periodontal–Brain Axis

**DOI:** 10.1007/s12640-026-00802-5

**Published:** 2026-07-16

**Authors:** Seyed Ebrahim Alavi, Hasan Ebrahimi Shahmabadi, Robert M. Love, Arun V. Kurumathur, Lavanya A. Sharma, Ajay Sharma

**Affiliations:** 1 School of Medicine and Dentistry, Griffith University, Gold Coast, QLD Australia; 2 Immunology of Infectious Diseases Research Center, Research Institute of Basic Medical Sciences, Rafsanjan University of Medical Sciences, Rafsanjan, Iran; 3 Department of Periodontics, School of Dentistry, Amrita University, Kochi, Kerala India

**Keywords:** Alzheimer’s disease, Periodontitis, Extracellular vesicles, Outer membrane vesicles, Neuroinflammation

## Abstract

Alzheimer’s disease (AD) is a progressive neurodegenerative disorder whose origins extend beyond the brain. Chronic periodontitis has emerged as a modifiable risk factor, and extracellular vesicles (EVs) have recently been proposed as important mediators of the periodontal–brain axis. Periodontal pathogens such as *Porphyromonas gingivalis* (*P. gingivalis*) release bacterial EVs enriched with virulence factors including gingipains, lipopolysaccharide, and regulatory RNAs. These vesicles can enter systemic circulation, interact with the blood–brain barrier, activate microglia, and trigger inflammatory signaling pathways such as NF-κB and NLRP3. These processes contribute to neuroinflammation, amyloid-β accumulation, and tau hyperphosphorylation, hallmarks of AD pathology. Host-derived EVs further contribute to this complex signaling network by facilitating intercellular communication and potentially propagating pathogenic proteins while also carrying protective molecules. Preclinical studies suggest that periodontal-derived vesicles can reach the hippocampus and impair cognition, while clinical studies have detected *P. gingivalis* DNA and gingipains in AD brain tissues. EV–associated biomarkers in blood or cerebrospinal fluid and engineered therapeutic vesicles represent promising tools for early diagnosis and intervention. Targeting oral microbial EVs may therefore offer novel avenues for AD prevention and therapy.

**Supplementary Information:**

The online version contains supplementary material available at https://doi.org/10.1007/s12640-026-00802-5.

## Introduction

Alzheimer’s disease (AD) is the most prevalent form of dementia, accounting for 60–80% of all cases worldwide (Inchingolo et al. 2025; R. Li et al. 2024; Popescu et al. 2024; Pruntel et al. 2024; Tang 2024). This progressive, irreversible neurodegenerative disorder is characterized clinically by memory loss, cognitive decline, and behavioral changes (Grahl et al. 2025; Popescu et al. 2024; Pruntel et al. 2024; Qiu et al. 2025). Pathologically, AD is defined by two hallmark features: the extracellular deposition of beta-amyloid (Aβ) peptides forming senile plaques and the intracellular aggregation of hyperphosphorylated Tau protein forming neurofibrillary tangles (NFTs) (Grahl et al. 2025; Subedi et al. 2022; Tang et al. 2023; Tang 2024; Wu et al. 2025a, b). With over 55 million individuals globally currently living with dementia, and projections indicating this number could surge to 75 million by 2030 and 152 million by 2050, AD represents a major global public health and socioeconomic challenge (Inchingolo et al. 2025; R. Li et al. 2024; Pruntel et al. 2024; Tang et al. 2023). While AD etiology is complex, involving genetic susceptibility (e.g., ApoE4) and various environmental and lifestyle risk factors, the precise pathophysiology remains inadequately understood (Inchingolo et al. 2025; Ivana Shawkatova et al. 2025a; Tang et al. 2023).

Periodontitis (PD), a chronic inflammatory disease affecting the supporting tissues of the teeth, has emerged as a significant and potentially modifiable risk factor for AD (Alavi et al. 2025a, b; Fadzli et al. 2024; Inchingolo et al. 2025; R. Li et al. 2024). PD is a highly prevalent condition, impacting over 50% of the adult US population, and increasing in severity with age (Elashiry et al. 2024; Inchingolo et al. 2025; Ivana Shawkatova et al. 2025a). It is associated with microbial dysbiosis characterized by the predominance of periodontal pathogens such as *Porphyromonas gingivalis* (*P. gingivalis*), *Tannerella forsythia* (*T. forsythia*), *Fusobacterium nucleatum* (*F. nucleatum*), and *Treponema denticola* (*T. denticola*) (Alavi et al. 2023a, b; Alavi et al. 2022a, b, 2023a, b, 2024a, b, c; Baker et al. 2024; Thomas et al. 2021). The presence of inflamed periodontal pockets provides a persistent source of bacteria and virulence factors that can enter the systemic circulation (Vellapandian et al. 2025).

This chronic systemic inflammation and the migration of pathogenic components from the oral cavity to the central nervous system (CNS) define the crucial connection known as the periodontal–brain axis (Lee et al. 2023; R. Li et al. 2024; Vellapandian et al. 2025). Established mechanistic hypotheses linking PD to AD pathology include the hematogenous dissemination of pathogens or toxins across a compromised blood–brain barrier (BBB), chronic systemic inflammation driving neuroinflammation through cytokine release (IL-1β, IL-6, TNF-α), and the direct neuroinvasion by pathogens like *P. gingivalis* and its potent virulence factors, particularly the cysteine proteases called gingipains (H.-L. Chen et al. 2023a, b; Fadzli et al. 2024; Huang et al. 2025; Inchingolo et al. 2025; Ivana Shawkatova et al. 2025a; Tang et al. 2023; Vellapandian et al. 2025; Wereszczyński et al. 2023; Wu et al. 2025a, b). The detection of *P. gingivalis* DNA and gingipains in the brains of AD patients provides compelling post-mortem evidence supporting this linkage (Elashiry et al. 2024; Inchingolo et al. 2025; R. Li et al. 2024; Ivana Shawkatova et al. 2025a; Tang et al. 2023; Wu et al. 2025a, b). 

Despite strong associations, a major knowledge gap persists concerning the precise mechanisms by which *P. gingivalis* or its components traverse the BBB and impact brain health, particularly since definitive evidence of whole, viable bacterial colonization in the brain is often lacking (Elashiry et al. 2024; Qiu et al. 2025; Ivana Shawkatova et al. 2025a). Extracellular vesicles (EVs) have recently been identified as efficient mediators of intercellular and interkingdom communication that may facilitate this translocation (Elashiry et al. 2024; Ivana Shawkatova et al. 2025a; Vellapandian et al. 2025; Wu et al. 2025a, b). EVs are nanosized, lipid bilayer vesicles actively secreted by cells, ranging from 10 to 300 nm (Lee et al. 2023; Tang 2024). They serve as vital long-distance carriers for genetic information, proteins, and virulence factors (Elashiry et al. 2024; Lee et al. 2023; Vellapandian et al. 2025). In this review, the term EVs is used as a general descriptor for membrane-bound vesicles released by cells. Host cell–derived vesicles originating from the endosomal pathway are referred to as exosomes, whereas vesicles produced by Gram-negative bacteria such as *P. gingivalis* are referred to as outer membrane vesicles (OMVs). Also, we distinguish between two biologically distinct vesicle populations involved in periodontal–brain communication. Bacterial EVs, particularly OMVs released by Gram-negative pathogens such as *P. gingivalis*, primarily function as carriers of virulence factors that contribute to inflammation and tissue damage. In contrast, host-derived EVs, including exosomes released by mammalian cells, participate in physiological intercellular communication and may exert either protective or pathogenic effects depending on their molecular cargo. For clarity, the following sections discuss these vesicle types separately.

*P. gingivalis*-derived OMVs, a subset of EVs, are abundant producers of virulence factors like gingipains and lipopolysaccharide (LPS) (Fan et al. 2023; Wu et al. 2025a, b). Recent studies demonstrate that host cell-derived exosome isolated from the gingiva of PD patients (PD exosomes) contain *P. gingivalis* antigens (RGP and Mfa-1) and inflammatory cytokines (IL-1β and IL-6) (Elashiry et al. 2024). Crucially, these PD exosomes were shown to penetrate the BBB in vitro and, when injected into the oral cavity of mice, penetrated the brain and localized with hippocampal microglial cells (Elashiry et al. 2024). The mechanisms involve the capacity of EVs, acting as “Trojan Horses,” to disrupt the BBB by compromising brain microvascular endothelial cell (BMEC) permeability, potentially through components like gingipains (Elashiry et al. 2024; Ivana Shawkatova et al. 2025a; Wu et al. 2025a, b).

This article is a structured narrative review synthesizing evidence from in vitro, in vivo, and clinical studies pertaining to the role of oral microbial EVs in AD. A narrative approach was chosen because the field is emerging, heterogeneous, and not yet suited for formal meta-analytic synthesis. While not a systematic review, we incorporated systematic principles—including predefined thematic categories and a comprehensive search of PubMed, Scopus, and Web of Science—to ensure breadth and rigor. We explicitly distinguish between empirical evidence and authors’ interpretations and critically evaluate methodological differences across studies to clarify the current limitations and controversies in the field. Prior reviews on periodontal–brain interactions vary considerably in methodological rigor, ranging from descriptive narrative summaries to systematic evidence syntheses with meta-analyses. This heterogeneity contributes to inconsistencies in reported conclusions. By highlighting these methodological differences throughout the manuscript, we aim to clarify which findings are robust, which remain speculative, and where future systematic evaluations are needed.

Also, the purpose of this review is to synthesize current evidence on the contribution of oral microbial EVs—particularly those derived from periodontal pathogens—to AD pathogenesis. The intended audience includes neuroscientists, microbiologists, periodontology researchers, and clinicians interested in neuroinflammation and host–microbe interactions. By integrating molecular, microbiological, and clinical perspectives, the review aims to make this emerging interdisciplinary field accessible to a broad scientific readership. To remain current, this review incorporates recent evidence from 2023 to 2025, including advances in exosomal miRNA profiling and microbial vesicle tracking in neurodegenerative models. Foundational studies from earlier decades are included where they remain essential for understanding periodontal pathogenesis, exosome biology, and AD mechanisms. Accordingly, this review does not aim to establish a direct causal relationship between periodontal disease and AD. Instead, it focuses on synthesizing evidence for specific mechanistic pathways—particularly microbial EVs and host exosome-mediated signaling, neuroinflammation, and amyloid/tau-associated processes—that may link chronic oral inflammation to Alzheimer’s pathogenesis. By concentrating on defined molecular and cellular mechanisms, this review seeks to clarify biologically plausible associations while acknowledging existing limitations in the field.

This manuscript is designed as a critical narrative review that synthesizes current evidence on the role of oral microbial EVs in AD pathogenesis. Although a structured literature search strategy was used to identify relevant publications, this review does not follow the formal methodological framework of a systematic or scoping review (e.g., PRISMA guidelines). Instead, studies were selected based on their relevance to extracellular vesicle (EV) biology, periodontal–brain communication, and mechanisms of neuroinflammation associated with AD. The structured search approach was used to improve transparency and ensure broad coverage of the literature while maintaining the interpretive flexibility characteristic of narrative reviews.

## Literature Search Strategy

A structured literature search was conducted using PubMed, Web of Science, and Scopus databases. The search covered studies published between January 2000 and March 2025, reflecting the emergence of EV biology and its relevance to neurodegenerative disease. Search terms included combinations of the following keywords: periodontal disease, oral microbiome, outer membrane vesicles, exosomes, extracellular vesicles, blood-brain barrier, neuroinflammation, beta-amyloid, tau phosphorylation, and AD. Boolean operators (AND/OR) were applied to refine searches. Reference lists of relevant review articles were manually screened to identify additional studies. Only articles published in peer-reviewed journals and available in English were considered. The detailed search strategy, including representative PubMed search terms and Boolean operators used for database retrieval, is provided in supplementary Table S1. Studies were selected based on their mechanistic relevance to exosome biology, periodontal–brain communication, and AD pathology. Priority was given to investigations that provided direct experimental evidence of vesicle-mediated transport, barrier permeability, neuroinflammatory signaling, or amyloid/tau modulation. When multiple studies addressed similar mechanisms, representative examples were cited to support narrative synthesis rather than exhaustive visual presentation, in keeping with best practices for critical review articles.

### Study Selection and Inclusion Criteria

To improve transparency in study selection, the inclusion criteria were defined using the PICOS framework (Population, Intervention/Exposure, Comparison, Outcomes, and Study design).

#### Population

Studies involving human subjects, animal models, or in vitro cellular systems relevant to periodontal disease, oral microbiota, EVs, and neurodegenerative processes were considered.

#### Intervention/Exposure

Studies examining periodontal disease, periodontal pathogens (particularly *P. gingivalis*), oral microbial EVs vesicles, or bacterial OMVs and their potential interactions with the CNS were included.

#### Comparison

Where applicable, studies comparing diseased versus healthy conditions, infected versus non-infected models, or vesicle-exposed versus control systems were considered.

#### Outcomes

Eligible studies reported outcomes related to AD–associated mechanisms, including neuroinflammation, BBB integrity, Aβ accumulation, tau phosphorylation, cognitive impairment, or EV–mediated signaling pathways.

#### Study Design

Relevant experimental and observational studies were included, encompassing in vitro studies, animal models, and human observational research. Review articles were also screened to identify additional relevant primary studies through reference lists.

#### Exclusion Criteria

Studies were excluded if they lacked mechanistic relevance to EVs, periodontal disease, or neurodegenerative processes; focused exclusively on unrelated systemic diseases; were case reports; or lacked sufficient methodological detail.

## Evaluation of Evidence Quality

Because this manuscript is designed as a critical narrative review rather than a formal systematic review, the quality of the included studies was evaluated qualitatively rather than through formal risk-of-bias scoring systems. Studies were interpreted based on several factors, including study design, methodological rigor, and relevance to mechanisms linking periodontal disease, EVs, and AD pathology.

Greater emphasis was placed on studies providing mechanistic evidence, particularly those demonstrating vesicle-mediated transport, BBB interactions, neuroinflammatory signaling, or modulation of amyloid and tau pathology. Findings supported by multiple experimental approaches—such as in vitro experiments combined with animal models or human observational data—were considered more robust.

Conversely, findings derived from single experimental systems or purely associative observations were interpreted cautiously and discussed within the context of their methodological limitations.

### The Periodontal–Brain Axis: Established Mechanisms

**Fig. 1:**
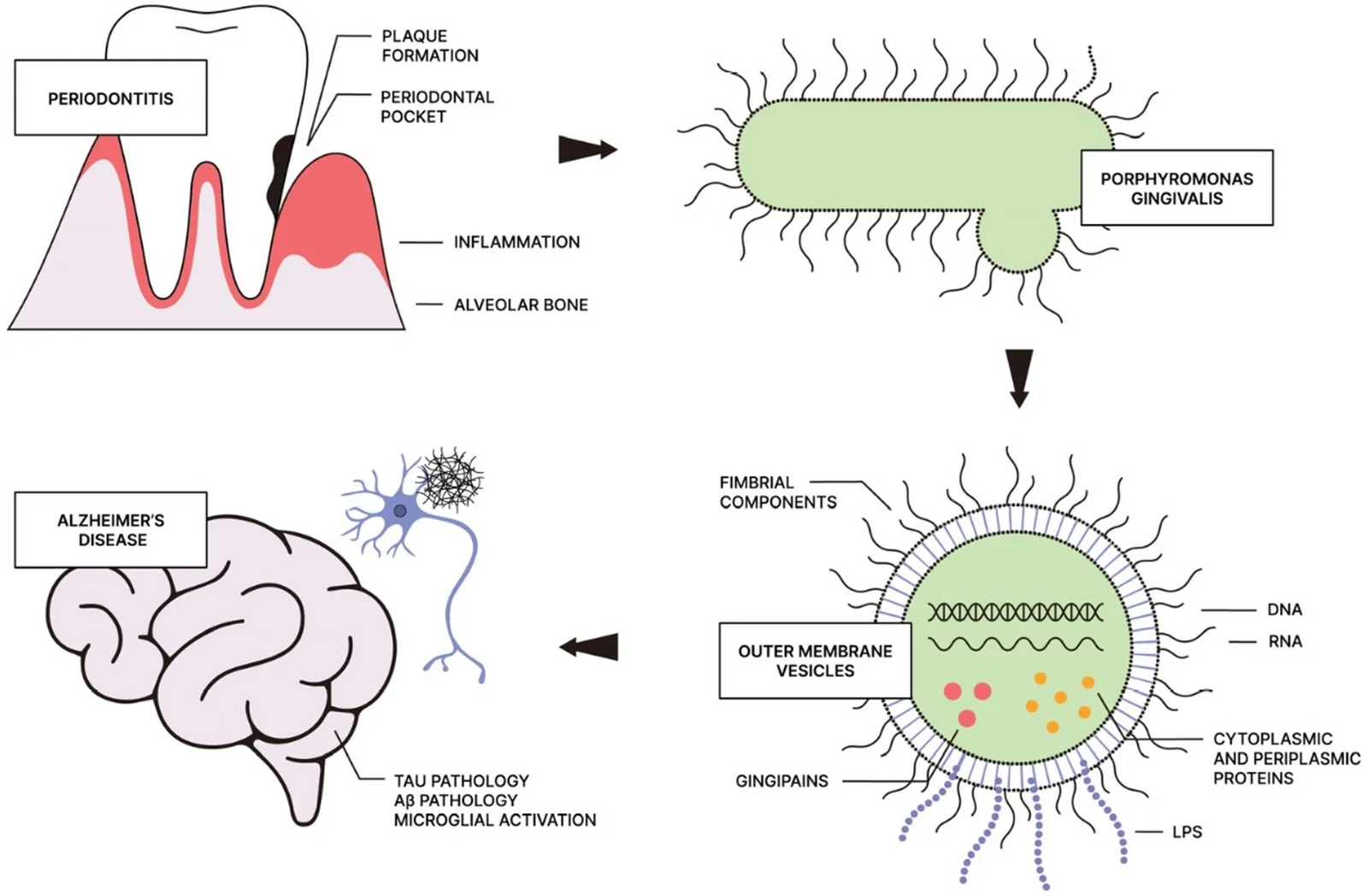
A schematic representation of *P. gingivalis* colonizing the subgingival pockets during periodontitis and disseminating its virulence factors through outer membrane vesicles to the brain, where they may play a role in the progression of Alzheimer’s disease pathology. This figure is reproduced with permission from ref (Ivana Shawkatova et al. 2025a). Copyright 2025 MDPI

The periodontal-brain axis is supported by pathological links between chronic PD and AD (Vellapandian et al. 2025). While classical models emphasize the systemic spread of whole oral pathogens (particularly *P. gingivalis*) or free soluble inflammatory products (Fig. 1), emerging evidence indicates that oral microbial EVs (OMVs) and host-derived exosomes act as the primary, highly efficient vectors mediating this axis. Rather than acting through a single pathway, OMVs influence AD pathology through a convergence of vascular, immune, and neuronal mechanisms, fundamentally modifying how periodontal virulence factors reach and perturb the brain (Fadzli et al. 2024; Vellapandian et al. 2025). Periodontal pathogens such as *P. gingivalis* have also been associated with a range of systemic diseases beyond oral inflammation, including gastrointestinal disorders, metabolic diseases, and cancer. Emerging evidence suggests that the oral microbiome may influence distant organ systems through inflammatory mediators, microbial metabolites, and EVs. Dysbiosis of the oral microbiota has been linked to intestinal inflammation, non-alcoholic fatty liver disease, and other systemic conditions that themselves have been associated with increased risk of neurodegenerative disorders. These interconnected disease processes highlight the potential role of oral microbial dysbiosis as part of a broader systemic network influencing chronic inflammatory diseases and neurodegeneration (Peng et al. 2022; Tian et al. 2024). In this regard, while these findings suggest a plausible mechanism through which microbial vesicles may influence neurodegenerative processes, the evidence remains largely indirect and requires further validation in well-controlled experimental and clinical studies.

### Hematogenous Dissemination of Oral Pathogens

While whole pathogens can enter the bloodstream through ulcerated periodontal pockets and degrade blood-brain barrier (BBB) tight junctions (e.g., occludin, claudin-5) (Ivana Shawkatova et al. 2025a), OMVs offer a distinct, stealthier conduit. Recent comparative studies highlight this functional divergence. Qiu et al. (2025) demonstrated that while live *P. gingivalis* bacteria cause severe systemic inflammation (splenomegaly, plasma IL-1β/TNFα) alongside BBB disruption, OMVs penetrate the brain and induce hippocampal neuroinflammation with minimal peripheral immune activation. Similarly, Bradley et al. (Bradley et al. 2025) showed that intravenous OMV exposure in experimental models reaches the brain, increases p-Tau (Thr231), and activates microglia without a broad systemic cytokine signature. Furthermore, Elashiry et al. (Elashiry et al. 2024) confirmed that orally generated, microbe-altered exosomes traffic directly from the gingiva to the brain carrying *P. gingivalis* antigens, confirming OMVs as stealth bloodborne disseminators of neuropathology.

### Systemic Inflammation and Cytokine Signaling

PD creates chronic local inflammation, historically thought to drive AD via the “spillover” of peripheral cytokines (IL-1β, IL-6, TNF-α) that cross the BBB to activate resident microglia (H.-L. Chen et al. 2023a; Fadzli et al. 2024). However, OMVs refine our understanding of this systemic-to-central inflammatory signaling. By encapsulating and protecting virulence factors within a lipid bilayer, OMVs can decouple peripheral cytokine readouts from central neurotoxic effects. As noted in the Bradley et al. (Bradley et al. 2025) study, the lack of systemic cytokine upregulation despite profound central glial activation suggests that exosomes can bypass peripheral immune surveillance to directly initiate neuroinflammation. This OMV-mediated decoupling is a critical distinction for interpreting human biomarker studies, explaining why central AD pathology may progress even when peripheral inflammatory markers appear muted.

Beyond EV–mediated mechanisms, circulating LPS derived from Gram-negative bacteria may also contribute to systemic inflammation and neurodegenerative processes. LPS can enter the circulation through microbial translocation from mucosal surfaces, including the oral cavity and gastrointestinal tract, where it interacts with plasma components such as LPS-binding protein, albumin, and lipoproteins. Elevated plasma LPS levels have been associated with systemic inflammatory responses and may influence BBB integrity, thereby facilitating neuroinflammatory signaling within the CNS. Disruption of BBB function may also interfere with peripheral Aβ clearance mechanisms, potentially contributing to amyloid accumulation and neurodegeneration. These findings suggest that microbial components such as LPS, in addition to EVs carrying virulence factors, may play complementary roles in linking peripheral microbial dysbiosis with AD pathology (An et al. 2022; Chaby 2004; Mohr et al. 2022; Sharma and James Martins 2023).

### Direct Neuroinvasion by Periodontal Pathogens

The detection of *P. gingivalis* DNA and gingipains in AD brains strongly implicates direct neuroinvasion (Ivana Shawkatova et al. 2025a; Tang et al. 2023; Wu et al. 2025a, b). While whole bacteria may theoretically migrate via retrograde axonal transport through cranial nerves (e.g., the trigeminal nerve), OMVs provide a highly plausible, efficient vehicle for this direct anatomical conduit. Vesicles carrying concentrated virulence factors (gingipains, Rgp, Mfa-1) can easily traffic along neural pathways. Once in vulnerable brain regions like the hippocampus, OMV-delivered gingipains directly cleave tau proteins, promoting hyperphosphorylation and the formation of NFTs (Elashiry et al. 2024; Qiu et al. 2025). Thus, OMVs mediate direct pathogenic effects and tau seeding without requiring overt, whole-cell bacterial colonization.

### Amyloid Cross-Seeding Mechanisms

The “antimicrobial protection hypothesis” suggests Aβ plaques form as an innate defense mechanism to trap migrating pathogens or their toxins (Elashiry et al. 2024). OMVs, which are highly enriched with lipopolysaccharides (LPS) and gingipains, act as potent triggers for this amyloid cross-seeding. Upon entering the central nervous system, OMV-derived *P. gingivalis* LPS activates the lysosomal cysteine protease Cathepsin B. This activation subsequently increases BACE1 activity and expression, exacerbating aberrant amyloid precursor protein (APP) processing and driving Aβ generation and deposition (R. Li et al. 2024; Tang et al. 2023). By delivering concentrated doses of amyloidogenic triggers directly to neural tissue, OMVs integrate oral infections into the fundamental biochemical cascade of AD.

### Strengths and Limitations of Current Evidence

The major strengths of current evidence include the compelling epidemiological data demonstrating a correlation between chronic PD and increased risk of cognitive decline and AD (Fadzli et al. 2024; Inchingolo et al. 2025; Ivana Shawkatova et al. 2025a). Furthermore, the molecular detection of *P. gingivalis* virulence factors (DNA and gingipains) in human AD brains strongly supports a causal link (Table 1) (Inchingolo et al. 2025; Ivana Shawkatova et al. 2025a; Tang et al. 2023). Animal studies successfully replicate AD-like pathologies (neuroinflammation, Aβ production) through chronic oral pathogen exposure (R. Li et al. 2024; Ivana Shawkatova et al. 2025a; Tang et al. 2023; Vellapandian et al. 2025). However, significant limitations exist. Crucially, conclusive evidence proving active colonization by viable, whole *P. gingivalis* bacteria in human brains is missing (Elashiry et al. 2024; Qiu et al. 2025; Ivana Shawkatova et al. 2025a). Most mechanistic findings rely heavily on preclinical models, which may not translate completely to the complexity of human AD (R. Li et al. 2024). High heterogeneity across human studies also impedes definitive, comparative conclusions (Pruntel et al. 2024).

**Table 1:** Established mechanisms linking periodontitis and Alzheimer’s disease

Mechanism	Key evidence	Strengths	Limitations
Hematogenous dissemination of pathogens/toxins	Pathogens (e.g., *P. gingivalis*) and virulence factors (LPS, gingipains) enter the bloodstream through ulcerated periodontal pockets, leading to bacteremia (H.-L. Chen et al. 2023a; Tang et al. 2023; Vellapandian et al. 2025). Detection of *P. gingivalis* DNA/LPS/gingipains in post-mortem AD brain tissues (Inchingolo et al. 2025; Ivana Shawkatova et al. 2025a; Tang et al. 2023).	Provides a clearly defined physiological route (systemic circulation) for periodontal products to reach the CNS (Vellapandian et al. 2025). Molecular components of the pathogen are detected directly in the target organ (Inchingolo et al. 2025; Ivana Shawkatova et al. 2025a).	Definitive evidence demonstrating active colonization by the whole, viable bacterium in human brain tissue is lacking (Elashiry et al. 2024; Qiu et al. 2025; Ivana Shawkatova et al. 2025a). Mechanisms rely on pre-existing BBB compromise (Tang et al. 2023).
Systemic inflammation and cytokine release	Chronic PD elevates systemic pro-inflammatory cytokines (IL-1β, IL-6, TNF-α) (H.-L. Chen et al. 2023a; Inchingolo et al. 2025; R. Li et al. 2024). These cytokines cross the BBB and activate microglia, initiating neuroinflammation, which drives Aβ/tau pathology (Fadzli et al. 2024; Inchingolo et al. 2025; Vellapandian et al. 2025; Wereszczyński et al. 2023).	Strong epidemiological evidence links periodontal inflammation markers (like CRP) to increased cognitive decline risk (Inchingolo et al. 2025; Ivana Shawkatova et al. 2025a). Accounts for the “spillover” effect of chronic disease (Inchingolo et al. 2025).	Inflammatory responses are non-specific and are shared mechanisms across multiple systemic diseases associated with AD (H.-L. Chen et al. 2023a; Inchingolo et al. 2025).
Direct neuroinvasion by ***P. gingivalis*** and gingipains	Gingipains are detected in AD brains and their loads correlate with the severity of tau pathology (Ivana Shawkatova et al. 2025a; Tang et al. 2023). Gingipains degrade BBB tight junction proteins (e.g., ZO-1, occludin), increasing permeability (Qiu et al. 2025; Ivana Shawkatova et al. 2025a; Wu et al. 2025a, b). Migration via retrograde axonal transport through cranial nerves (e.g., trigeminal nerve) is proposed (Vellapandian et al. 2025; Wereszczyński et al. 2023).	Identifies a potent enzymatic mechanism that directly impacts core AD pathology (tau protein cleavage) (Ivana Shawkatova et al. 2025a; Wu et al. 2025a, b). Provides anatomical paths (cranial nerves) bypassing the BBB (Vellapandian et al. 2025).	Mechanisms of neural migration and direct invasion remain largely based on preclinical or in vitro models (Vellapandian et al. 2025; Wu et al. 2025a, b).
Amyloid cross-seeding hypothesis	Exposure to *P. gingivalis* components (LPS, etc.) promotes Aβ accumulation and deposition via aberrant APP processing (Ivana Shawkatova et al. 2025a; Tang et al. 2023; Vellapandian et al. 2025). Cathepsin B activation, induced by *P. gingivalis* LPS, plays a critical role in increasing Aβ generation (R. Li et al. 2024; Tang et al. 2023; Wu et al. 2025a, b). Aβ is theorized to be an antimicrobial peptide reacting to microbial threat (antimicrobial protection hypothesis) (Elashiry et al. 2024; R. Li et al. 2024).	Integrates the oral infection directly into the fundamental biochemical process of AD pathology (Aβ deposition) (Vellapandian et al. 2025; Wu et al. 2025a, b). Supported by a plausible evolutionary function for Aβ (R. Li et al. 2024).	Primary mechanism demonstrated in animal and cellular models; translating the quantitative significance to human sporadic AD is challenging (R. Li et al. 2024).

LPS: lipopolysaccharides; DNA: deoxyribonucleic acid; AD: Alzheimer’s disease; CNS: central nervous system; BBB: blood-brain barrier; Aβ: beta-amyloid; APP: amyloid precursor protein; CRP: C-reactive protein

The major strength of the current literature is the molecular detection of *P. gingivalis* virulence factors in human AD brains, coupled with animal models that successfully replicate AD-like pathologies using OMV exposure. However, because conclusive evidence proving active colonization by viable, whole bacteria in human brains remains elusive (Elashiry et al. 2024; Qiu et al. 2025; Ivana Shawkatova et al. 2025a), OMVs emerge as the most biologically plausible vector. The primary limitation moving forward is methodological: translating these mechanistic findings requires advanced techniques to definitively isolate, track, and distinguish microbe-derived OMVs from host-derived EVs in human clinical samples.

### Extracellular Vesicle Biology in Host–Microbe Communication

To understand how these mechanisms are executed, it is essential to distinguish the biological nature of the vesicles involved. EVs are nanosized membrane structures (30–150 nm) that mediate complex intercellular communication locally and systemically (Liang et al. 2023; Sun and Chen 2024). In the context of the periodontal-brain axis, recent studies highlight two distinct but synergistic vesicular populations:

1. **Host-derived EVs**: Infection by *P. gingivalis* triggers a massive (≈ 10²-fold) increase in the release of EVs from host epithelial cells, which carry altered, disease-associated cargo (Gegout et al. 2025).
2. **Microbe-derived OMVs**: Conversely, bona fide bacterial OMVs are secreted directly by pathogens like *P. gingivalis* (Fan et al. 2023). Both host-derived exosomes and microbial OMVs function as discrete effectors capable of traversing biological barriers intact, making them the primary suspects in the systemic dissemination of periodontal AD pathology.

### Definition and Classification of Extracellular Vesicles

EVs are nanosized membrane vesicles secreted by cells (Daksh et al. 2025). Based on their biogenesis, size, content, and function, EVs are classified into microvesicles, apoptotic bodies, and exosomes (Daksh et al. 2025). Exosomes contents contribute in material movement and information exchange across the body, and they are widely found in biological fluids (Liang et al. 2023; Sun and Chen 2024). The studies collectively distinguish host-derived EVs (cellular exosomes/EVs) from microbe-derived vesicles (bacterial OMVs). Gegout et al. (2025) focus on epithelial cell–derived EVs produced after *P. gingivalis* infection, showing a massive (≈ 102-fold) increase in EV release and altered cargo. Fan et al. (2023) and Xie et al. (2023a, b) study bona fide bacterial OMVs from *P. gingivalis* and *Helicobacter pylori* (*H. pylori*), respectively, highlighting OMVs as discrete microbial effectors able to traffic across barriers and interact with host cells.

### Biogenesis and Release of Extracellular Vesicles

**Fig. 2:**
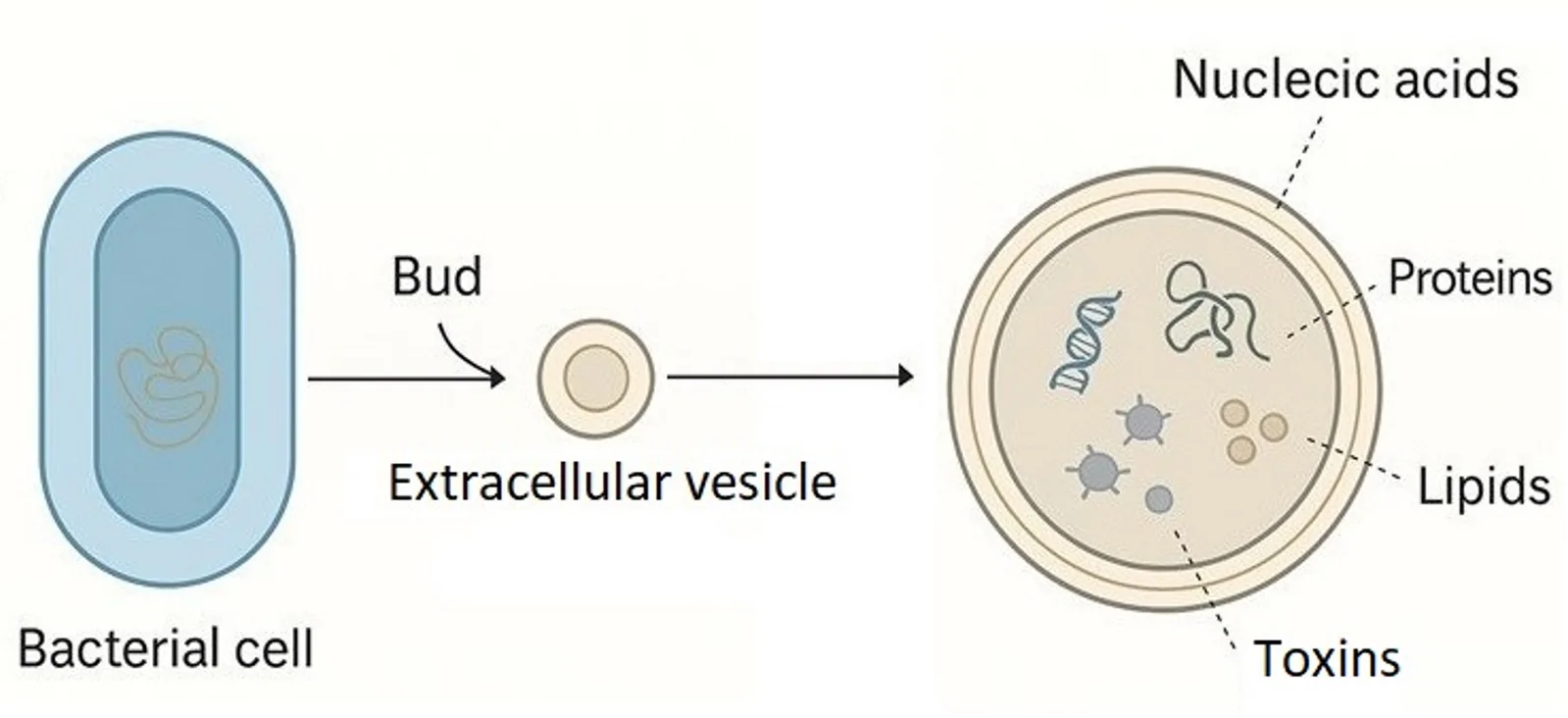
Biogenesis and cargo composition of bacterial extracellular vesicles (EVs). Schematic representation of EV formation from periodontal bacteria. In Gram-negative bacteria, EVs may include outer membrane vesicles, which bud from the outer membrane and encapsulate diverse cargo, including proteins such as gingipains, lipopolysaccharide, nucleic acids, and lipids. In broader terms, bacterial EVs serve as carriers of pathogenic molecules, enabling long-range host–microbe communication and potential involvement in AD pathogenesis

The biogenesis of exosomes begins with the inward budding of late endosomes to generate intraluminal vesicles (ILVs) within MVBs (Liang et al. 2023). MVBs are considered the progenitors of exosomes (Sun and Chen 2024). Exosomes are released into the extracellular space when the MVBs fuse with the plasma membrane, releasing the ILVs via exocytosis (Liang et al. 2023; Sun and Chen 2024). In pathological conditions like AD, the accumulation of APP cleavage in MVBs is observed, indicating that this biogenesis pathway plays a role in disease progression (Liang et al. 2023; Sun and Chen 2024). The biogenesis and release of exosomes are investigated in various studies. Gegout et al. (2025) demonstrate infection-driven upregulation of canonical EV secretion from oral epithelial cells, implying host biogenesis pathways are hijacked during dysbiosis. By contrast, Fan et al. (Fan et al. 2023) and Xie et al. (2023a, b) characterize OMVs as constitutive bacterial outputs containing msRNAs or proteins that are packaged during bacterial membrane blebbing—functionally analogous to exosomes but distinct in origin and molecular content. Figure 2 summarizes the biogenesis and cargo composition of bacterial EVs, with Gram-negative periodontal pathogens such as *P. gingivalis* producing OMVs as a defined subtype of bacterial EV.

### Cargo Composition of Bacterial and Host-Derived Vesicles

The molecular cargo of EVs varies depending on their cellular origin. Host-derived EVs, including exosomes released by mammalian cells, typically contain nucleic acids, proteins, and lipids involved in intercellular signaling and regulation (Liang et al. 2023; Sun and Chen 2024). Their cargo frequently includes messenger RNAs, microRNAs, long non-coding RNAs, and DNA fragments, as well as membrane-associated proteins such as CD9, CD63, CD81, and TSG101. Lipid components of host-derived EV membranes are enriched in phospholipids, sphingolipids, and ceramides, which contribute to vesicle stability and cellular uptake. In neurodegenerative diseases, host-derived EVs may also transport pathological proteins such as Aβ and tau, potentially contributing to the propagation of disease-related signaling within the CNS (Liang et al. 2023; Sun and Chen 2024).

In contrast, bacterial EVs, particularly those produced by Gram-negative periodontal pathogens, contain cargo that reflects bacterial virulence and host–microbe interactions. These vesicles frequently carry components such as LPS, outer membrane proteins, enzymes, toxins, and bacterial nucleic acids (Butler et al. 2024; S. Chen et al. 2023a; Elashiry et al. 2024; Fadzli et al. 2024). For example, EVs released by *P. gingivalis* may contain virulence factors including gingipains, fimbrial proteins, and immunomodulatory molecules capable of influencing host immune responses. These vesicles function as efficient delivery systems that transport bacterial components to host tissues, facilitating microbial communication, immune modulation, and systemic dissemination of pathogenic signals (Butler et al. 2024; S. Chen et al. 2023a; Elashiry et al. 2024; Fadzli et al. 2024).

Because of these distinct cargo profiles, bacterial EVs and host-derived EVs may influence AD pathology through different but potentially complementary mechanisms, including immune activation, neuroinflammatory signaling, and modulation of amyloid and tau pathways (Butler et al. 2024; Fadzli et al. 2024; Lee et al. 2023; Liang et al. 2023; Sun and Chen 2024).

### Host-Derived Exosomes Vs. Microbial-Derived Extracellular Vesicles

Host-derived exosomes are typically defined as small EVs released by various mammalian cells, including those in the CNS like neurons and glial cells (Liang et al. 2023; Sun and Chen 2024; H. Wu et al. 2025a, b). Microbial-derived EVs, particularly those produced by Gram-negative bacteria such as *P. gingivalis*, are distinct and known as OMVs (Fan et al. 2023; Lundergan et al. 2024; Qiu et al. 2025). OMVs bud directly from the bacterial outer membrane, are highly inflammatory, and range in size from 50 to 400 nm (Fan et al. 2023). They act as important delivery systems for bacterial virulence factors, including LPS, gingipains, and regulatory nucleic acids, enabling microbial communication with host cells and contributing to immune modulation and tissue damage (Fan et al. 2023; H. Y. Kim et al. 2024; Lee et al. 2023; Lundergan et al. 2024).

### Roles in Intercellular Communication and Disease Pathways

Exosomes act as dynamic carrier vehicles, facilitating material and information exchange by transporting cargo to recipient cells through internalization mechanisms such as phagocytosis or membrane fusion (Liang et al. 2023; Liu and Geng 2024). In disease, exosomes play a dual role (Daksh et al. 2025; Liang et al. 2023; Liu and Geng 2024; Sun and Chen 2024). They propagate AD pathology by transferring misfolded proteins like Aβ and tau to healthy neurons, accelerating their death (Liang et al. 2023; Sun and Chen 2024). Conversely, exosomes derived from healthy cells can promote Aβ clearance or deliver protective enzymes like neprilysin and insulin-degrading enzymes (Liang et al. 2023; Sun and Chen 2024). Additionally, exosomes are major natural nanocarriers of harmful inflammatory chemicals, strongly influencing neuroinflammation and oxidative stress in AD (Sun and Chen 2024). Furthermore, the collective evidence highlights exosomes and microbial OMVs as sophisticated couriers that bridge oral dysbiosis with systemic and neurodegenerative outcomes. Gegout et al. (2025) show that *P. gingivalis* infection drives oral epithelial cells to release EVs enriched in pro-inflammatory miRNAs, which in turn stimulate naïve epithelial cells to secrete TNF-α and IL-1β and increase metabolic activity. This creates a positive-feedback loop of epithelial activation and local tissue injury that can amplify periodontal inflammation and potentially “prime” distal immune responses.

Fan et al. (2023) extend this paradigm to bacterial vesicles themselves. Their *P. gingivalis* OMVs are readily endocytosed by human periodontal ligament cells, where the bacterial msRNA sRNA45033 directly targets the chromatin regulator CBX5. This epigenetic interference elevates p53-dependent apoptosis and up-regulates NLRP3 inflammasome components, producing both cell death and a robust inflammatory cytokine milieu. The ability of a single small RNA within OMVs to reprogram host gene expression underscores a precise, non-canonical communication channel that can reshape host cell fate. Xie et al. (2023a, b) reveal that OMVs from *H. pylori* move beyond local tissues, traversing biological barriers to reach the brain. Once internalized by astrocytes, they trigger complement C3–C3a receptor signalling, recruiting microglia and promoting synaptic pruning and loss of the presynaptic marker SYP and postsynaptic PSD-95. This cascade culminates in impaired long-term potentiation and cognitive decline, directly linking a gut-derived vesicle signal to Alzheimer-like pathology. Cao et al. (2025) provide an indirect but clinically relevant layer: by altering the oral microbiota with chlorhexidine and reducing *P. gingivalis* abundance, the upstream production of both host inflammatory EVs and microbial OMVs could be curtailed, suggesting that modulation of the oral ecosystem might attenuate these pathogenic communication routes.

Together, these findings portray exosomes and OMVs as dual agents of intercellular dialogue—host EVs amplifying inflammatory networks and microbial vesicles delivering targeted nucleic acids or proteins that rewire host immunity, epigenetics, and neuroglial interactions—thereby offering multiple mechanistic bridges between periodontal infection and AD progression.

#### Microbial Extracellular Vesicles in Alzheimer’s Disease Pathogenesis

This section synthesizes evidence on the ability of oral microbial EVs to influence CNS pathways relevant to AD. We organize the findings around five dominant themes: neuroinflammatory responses, amyloidogenic signaling, synaptic dysfunction, barrier permeability, and microbial–host molecular interactions. Oral microbial EVs, predominantly OMVs from periodontal pathogens, serve as key mediators in the link between PD and AD (Lundergan et al. 2024; Qiu et al. 2025; Wereszczyński et al. 2023). The major periodontal pathogen, *P. gingivalis*, produces OMVs containing virulence factors, including gingipains and LPS, which have been detected in AD brain tissue (Aina Oluwafemi and Ojini Kelechi 2025; Lundergan et al. 2024; Wereszczyński et al. 2023). These OMVs are capable of crossing biological barriers to enter the CNS (Koukoulis et al. 2024; Lundergan et al. 2024). Once there, they incite a neuroinflammatory response by activating glial cells, promoting Aβ accumulation, and contributing to neuronal damage and cognitive decline (Lundergan et al. 2024; Rajasekaran et al. 2024; Wereszczyński et al. 2023).

### OMV-Mediated Delivery of Virulence Factors

OMVs from *P. gingivalis* are loaded with potent virulence factors, particularly gingipains and LPS (Fan et al. 2023; Lundergan et al. 2024; Qiu et al. 2025). These specific bacterial components have been identified in the brain tissue and cerebrospinal fluid (CSF) of human AD patients (Aina Oluwafemi And Ojini Kelechi 2025; Lundergan et al. 2024). *P. gingivalis* OMVs induce neurotoxicity and cognitive dysfunction in animal models (Koukoulis et al. 2024; Qiu et al. 2025). Specifically, they trigger the NLRP3 inflammasome and induce neuroinflammation and tau phosphorylation (Qin et al. 2025; Qiu et al. 2025). Furthermore, gingipains enriched within OMVs can degrade tight junction proteins, leading to increased BBB permeability and enhanced access to the CNS (Qiu et al. 2025). Farrugia et al. (2020) suggested that OMVs from wild-type *P. gingivalis* (strain W83) disrupt endothelial integrity in a gingipain-dependent fashion. OMVs carrying surface gingipains cleaved the adhesion molecule PECAM-1 on human microvascular endothelial cells and increased dextran permeability, producing vascular pathology in a zebrafish model. These findings highlight a mechanism by which *P. gingivalis* EVs compromise the BBB and initiate vascular inflammation, providing a plausible entry route and neurotoxic trigger for AD. However, many of these findings are derived from animal models, and the extent to which these mechanisms translate to human AD remains uncertain.

#### Other Periodontal Pathogens (T. denticola, T. forsythia)

Besides *P. gingivalis*, other key periodontal pathogens, including *T. denticola* and *T. forsythia*, members of the Socransky’s Red Complex, are strongly associated with PD and implicated in AD progression (Gegout et al. 2025; Lundergan et al. 2024; Rajasekaran et al. 2024). Molecular and immunological evidence of oral *Treponema* species has been detected in human brain tissue, suggesting that these bacteria or their components, potentially encapsulated within OMVs, utilize neural pathways, such as the trigeminal ganglia, to reach the CNS (Aina Oluwafemi And Ojini Kelechi 2025; Lundergan et al. 2024; Rajasekaran et al. 2024). The altered oral microbiome composition observed in AD patients features these specific pathogens (Rajasekaran et al. 2024). Moreover, the genus *Treponema*, along with *P. gingivalis*, has been identified as a key target for intervention strategies aimed at slowing AD progression (Cao et al. 2025). Wu et al. (2022) showed that oral infection with *T. denticola* causes hippocampal neuronal apoptosis in mice via Aβ accumulation. Su et al. (2021) confirmed that *T. denticola* enters the brain and directly elevates Aβ_1_–40/_1_–42 by activating β- and γ-secretases, an effect blocked by respective inhibitors. Although EVs were not isolated in these studies, the data implicate *T. denticola* products—potentially OMVs—as upstream drivers of amyloidogenic pathways comparable to *P. gingivalis*, strengthening the concept that diverse periodontal pathogens converge on Aβ dysregulation.

#### Blood–Brain Barrier Disruption by Microbial Vesicles

The systemic dissemination of oral microbial EVs relies on their ability to cross the BBB (Koukoulis et al. 2024; Lundergan et al. 2024). OMVs from periodontal pathogens, such as *P. gingivalis* and *H. pylori*, have been demonstrated to successfully transit biological barriers and reach the brain (Koukoulis et al. 2024; Lundergan et al. 2024). Studies show that *P. gingivalis* OMVs can impair the integrity of tight junction proteins in the BBB (Qiu et al. 2025). The degradation of tight junction proteins, such as ZO-1 and occludin, is specifically attributed to gingipains carried within the OMVs, resulting in increased vascular and BBB permeability (Koukoulis et al. 2024; Qiu et al. 2025). Additionally, bacterial extracellular RNAs carried within OMVs from periodontopathogens have also been shown to cross the BBB in mice (Fan et al. 2023; Gegout et al. 2025; Koukoulis et al. 2024).

Han et al. (2019) and Ha et al. (2020) provided direct evidence that OMVs from *Aggregatibacter actinomycetemcomitans* (*A*. *actinomycetemcomitans*) traverse the BBB and accumulate in meningeal macrophages and microglia. Live imaging in CX3CR1-GFP mice revealed earlier uptake by meningeal macrophages than cortical microglia, while OMV-derived exRNAs—but not DNA—activated IL-6 and NF-κB signalling. These results confirm that microbial EVs can circulate systemically, breach the BBB, and deliver functional RNA cargo to central immune cells.

#### Neuroinflammation and Microglial Activation

Microbial EVs mediate complex pathological crosstalk within the CNS by interacting with neuronal and glial cells (Lundergan et al. 2024; Wereszczyński et al. 2023). *P. gingivalis* OMVs and their virulence factors, once delivered to the brain, trigger a severe neuroinflammatory cascade (Lundergan et al. 2024; Wereszczyński et al. 2023). The OMVs are taken up by glial cells, specifically astrocytes, contributing to subsequent inflammatory responses (Koukoulis et al. 2024; Lundergan et al. 2024). Microglial activation is a characteristic response, where microglia produce exaggerated pro-inflammatory mediators that stimulate Aβ production and induce neurotoxic changes (Lundergan et al. 2024; Wereszczyński et al. 2023). For example, studies show that *P. gingivalis* OMVs activate the NLRP3 inflammasome pathway in the brain, contributing directly to neuroinflammation and tau pathology (Qin et al. 2025; Qiu et al. 2025). Wei et al. (2020a, b) extended these findings by showing that OMVs increase BBB permeability, activate astrocytes and microglia, and drive tau hyperphosphorylation through GSK-3β in the hippocampus, culminating in cognitive deficits. Together with Ha et al. ’s cytokine induction (Ha et al. 2020) and Farrugia’s endothelial disruption (Farrugia et al. 2020), these data illustrate a multi-cellular dialogue in which microbial vesicles engage endothelial, microglial, and astroglial targets to propagate neuroinflammation and AD-like pathology.

### Systemic Inflammatory Roles of Microbial Extracellular Vesicles

The periodontal disease generates systemic inflammation as periodontal pathogens and their virulence factors, including components encapsulated in OMVs, enter the circulation (Lundergan et al. 2024; Qiu et al. 2025; Wereszczyński et al. 2023). Microbial EVs act as effective shuttles for inflammatory cargo like LPS and gingipains, triggering peripheral immune responses (Kondaveeti et al. 2022; Koukoulis et al. 2024). This chronic systemic inflammation influences the brain by activating microglia across the compromised BBB (Table 2) (Lundergan et al. 2024; Wereszczyński et al. 2023). Exposure to LPS from *P. gingivalis* induces neuroinflammation and stimulates the production and accumulation of Aβ in mice (Qiu et al. 2025). Therefore, the inflammatory response originating from the oral pathogenic microbiota and mediated by their OMVs is viewed as a crucial mechanism contributing to AD etiology (Kondaveeti et al. 2022; Lundergan et al. 2024). Across studies, microbial OMVs consistently elicit pro-inflammatory cytokines (IL-1β, IL-6, TNF-α) and activate NF-κB. While *P. gingivalis* OMVs emphasize proteolytic vascular damage, *A. actinomycetemcomitans* vesicles highlight exRNA-mediated immune activation (Ha et al. 2020)d *denticola* infection links periodontal inflammation to amyloidogenesis (Su et al. 2021; Wu et al. 2022). Microbial EVs have also been shown to influence systemic inflammatory pathways beyond the oral cavity. For example, EVs derived from periodontal pathogens can carry virulence-associated molecules that activate host immune signaling pathways and contribute to inflammatory responses in distant tissues. Recent work by Fang et al. (2024) further demonstrated that bacterial EVs can modulate inflammatory responses and influence host tissue remodeling, highlighting their potential role in systemic disease mechanisms beyond periodontal tissues. Collectively, these mechanisms converge on systemic inflammation, BBB compromise, and neuronal injury, positioning oral microbial EVs as potent, multifaceted mediators of Alzheimer’s pathogenesis. Although several mechanisms have been proposed—including neuroinflammation, amyloidogenic signaling, epithelial barrier disruption, and systemic dissemination—the strength of evidence varies considerably. Some studies provide direct mechanistic data using neuronal cultures or transgenic AD mice, whereas others infer causality based on correlative EVs markers detected in saliva or serum. Nonetheless, a significant portion of these results come from animal studies, and it is unclear how well these mechanisms apply to human AD. Also, since the methodological designs differ substantially, it remains unclear whether EVs act as primary drivers of neurodegeneration or secondary amplifiers of pre-existing inflammation. These discrepancies highlight the need for standardized isolation protocols and mechanistic assays to delineate causal relationships.

**Table 2:** Studies on oral microbial extracellular vesicles in Alzheimer’s disease models

Pathogen	Extracellular vesicle cargo	Model type	Observed effects	Ref.
*P. gingivalis*	Gingipains, LPS, etc. (OMVs)	Mice (Model not specified, potentially chronic injection)	Triggered NLRP3 inflammasome, induced neuroinflammation, tau phosphorylation, and memory dysfunction.	(Qin et al. 2025; Qiu et al. 2025)
*P. gingivalis*	OMVs	Middle-aged wild-type mice (oral gavage)	Reached the brain, impaired expression of tight junction proteins, induced inflammation, and impaired learning and memory.	(Koukoulis et al. 2024)
*P. gingivalis*	Extracellular RNAs (in OMVs)	Mice (tail vein injection)	Extracellular RNAs crossed the BBB.	(Fan et al. 2023; Gegout et al. 2025; Koukoulis et al. 2024)
*H. pylori*	OMVs	Alzheimer’s model mice (oral delivery)	Taken up by astrocytes, increased plaque load, inflammation, neuronal dysfunction, and accelerated cognitive decline.	(Koukoulis et al. 2024)
*H. pylori*	OMVs	Mice (venous and oral administration)	Trafficked to the brain, activated astrocytes, and caused neuronal damage.	(Koukoulis et al. 2024)

OMVs: outer membrane vesicles; BBB: blood-brain barrier

### Molecular Pathways Linking Extracellular Vesicles to AD Pathogenesis

Oral microbial OMVs are critical mediators linking periodontal disease to AD pathogenesis (Butler et al. 2024; Ivana Shawkatova et al. 2025a). OMVs from pathogens like *P. gingivalis* encapsulate virulence factors, including gingipains and LPS, enabling their translocation across the BBB (Butler et al. 2024; Ivana Shawkatova et al. 2025a). Once in the CNS, these microbial vesicles initiate key AD neuropathologies (Ivana Shawkatova et al. 2025a, b). Molecular pathways involved include the triggering of NF-κB and NLRP3 inflammasome signaling, promoting neuroinflammation, and disrupting Aβ and tau homeostasis (Butler et al. 2024; S. Chen et al. 2023a; Ivana Shawkatova et al. 2025a). Furthermore, EVs participate in the inter-neuronal spread or clearance of toxic Aβ and hyperphosphorylated tau proteins (Sun and Chen 2024).

## NF-κB Activation and Neuroinflammation

Oral microbial EVs, often characterized as OMVs, are potent activators of neuroinflammation via the NF-κB pathway (Butler et al. 2024; Ivana Shawkatova et al. 2025a). *P. gingivalis* OMVs contain virulence factors that trigger NF-κB signaling upon reaching the CNS (Ivana Shawkatova et al. 2025a). Specifically, *P. gingivalis* LPS drives microglia polarization to the proinflammatory M1 phenotype through the TLR2/4-mediated NF-κB pathway (Ivana Shawkatova et al. 2025a). Similarly, *A*. *actinomycetemcomitans* bacterial membrane vesicles (BMVs) activate NF-κB in macrophages and microglia, leading to increased expression of pro-inflammatory cytokines, such as TNF-α and IL-6 (Butler et al. 2024). This inflammatory response is a characteristic feature of AD pathogenesis (Butler et al. 2024; Ivana Shawkatova et al. 2025a). In detail, Han et al. (2019) revealed that small RNAs packaged within *A*. *actinomycetemcomitans* OMVs mimic eukaryotic miRNAs and enter host RNA-induced silencing complexes. These exRNAs activated TLR8, increased TNF-α, and enhanced NF-κB phospho-p65 in macrophage-like U937 cells; RNase treatment sharply reduced this effect. Ha et al. (2020) extended these findings to microglia (BV2 cells), showing that only the RNA cargo—not DNA—drove IL-6 secretion and NF-κB activation. Together these studies show that microbial EVs directly engage innate immune pathways, providing a mechanistic link between oral infection, chronic neuroinflammation, and AD.

### Host-Derived Extracellular Vesicles in Neurodegeneration

#### Extracellular Vesicle-Mediated Propagation of Amyloid and Tau Pathology

EVs and OMVs play a key role in propagating the characteristic AD features: Aβ accumulation and tau hyperphosphorylation (Ivana Shawkatova et al. 2025a; Sun and Chen 2024). *P. gingivalis* OMVs promote tau phosphorylation and induce Aβ pathology in neurons, both in vitro and in vivo (Butler et al. 2024; S. Chen et al. 2023a; Ivana Shawkatova et al. 2025a). Specifically, gingipains carried by OMVs contribute to Aβ production and facilitate tau cleavage and phosphorylation (Ivana Shawkatova et al. 2025a). Host-derived exosomes are implicated in the spread of Aβ and pathological tau throughout the brain (Sun and Chen 2024; Zhao et al. 2023). The APP cleavage products (Aβ) are packaged into exosomes during the endosomal pathway (Sun and Chen 2024; Zhao et al. 2023). Although not focused on tau or amyloid directly, the Han (Han et al. 2019)/Ha (Ha et al. 2020) data establish upstream inflammatory signaling that can potentiate tau kinases. Wei et al. (2020a, b) previously showed OMVs induce GSK-3β–dependent tau phosphorylation in hippocampus, aligning with this pro-inflammatory cascade and supporting a vesicle-driven route to canonical AD lesions.

##### Protective Roles of Neuronal Extracellular Vesicles

Exosomal microRNAs (miRNAs) are crucial modulators of neuronal health and dysfunction in AD (Feng et al. 2025; Lin et al. 2025). Microbial OMVs, such as those from *P. gingivalis*, induce neurotoxicity, impair memory, and cause synaptic dysfunction, potentially by suppressing NMDAR/BDNF signaling in the hippocampus (Butler et al. 2024; Ivana Shawkatova et al. 2025a). The hyperphosphorylation of tau protein, which can be promoted by the absence of miR-124-3p, results in neurodegenerative changes (Lin et al. 2025). Conversely, specific host exosomes offer neuroprotection; for example, mesenchymal stem cell (MSC)-derived exosomal miR-223 guards against neuronal apoptosis (Feng et al. 2025; Sun and Chen 2024), while miR-124-3p enriched microglial exosomes mitigate neurodegeneration (Lin et al. 2025). Aβ-linked, ceramide-enriched exosomes also trigger caspase-mediated neuronal cell death (Sun and Chen 2024). Duan et al. (2020) suggested that MSC-derived exosomes enriched in miR-146a-5p protect neurons by downregulating IRAK1 and NFAT5, reducing apoptosis and dampening microglial M1 polarization. Likewise, Wei et al. (2020a, b) identified MSC-exosomal miR-223 as a potent anti-apoptotic factor via the PTEN–PI3K/Akt pathway. These protective host exosomal miRNAs counterbalance the pro-inflammatory microbial signals, highlighting therapeutic potential.

#### Oxidative Stress and Mitochondrial Dysfunction

Oxidative stress and mitochondrial dysfunction are strongly linked to AD pathogenesis (Ivana Shawkatova et al. 2025a; Sighencea et al. 2024; Sun and Chen 2024). *P. gingivalis* LPS contributes to neurotoxicity by exacerbating reactive oxygen species (ROS) production, inducing oxidative stress, and causing mitochondrial dysfunction (Ivana Shawkatova et al. 2025a, b). In AD, Aβ plaques trigger cellular oxidative stress responses (Sun and Chen 2024). Moreover, astrocyte-derived exosomes (ADEs) enriched with ceramide and linked to Aβ pathology can be transferred to mitochondria, leading to mitochondrial clustering and increased DRP1 levels (Sun and Chen 2024). Controlling specific exosomal microRNAs may mitigate these effects, as inhibiting miR-125b-5p is suggested to reduce ROS levels and protect against oxidative stress (Feng et al. 2025). Du et al. (2021) showed ADEs lessen oxidative stress in hypoxic-ischemic rat brains by boosting antioxidant enzymes (SOD, GSH-Px, CAT) and lowering MDA, TNF-α, and IL-1β. Li et al. (B. Li et al. 2024) reported that neural stem cell–derived exosomes enhance mitochondrial biogenesis through the SIRT1–PGC1α axis, increasing NRF1 and COXIV in AD mice while reducing astrocyte activation. These data emphasize the dual role of host exosomes: mitigating oxidative damage and restoring mitochondrial homeostasis disrupted in AD. However, many of these findings are based on animal experiments, and it is uncertain to what degree these mechanisms are relevant to human AD.

## Interaction between Microbial and Host Vesicles

**Fig. 3:**
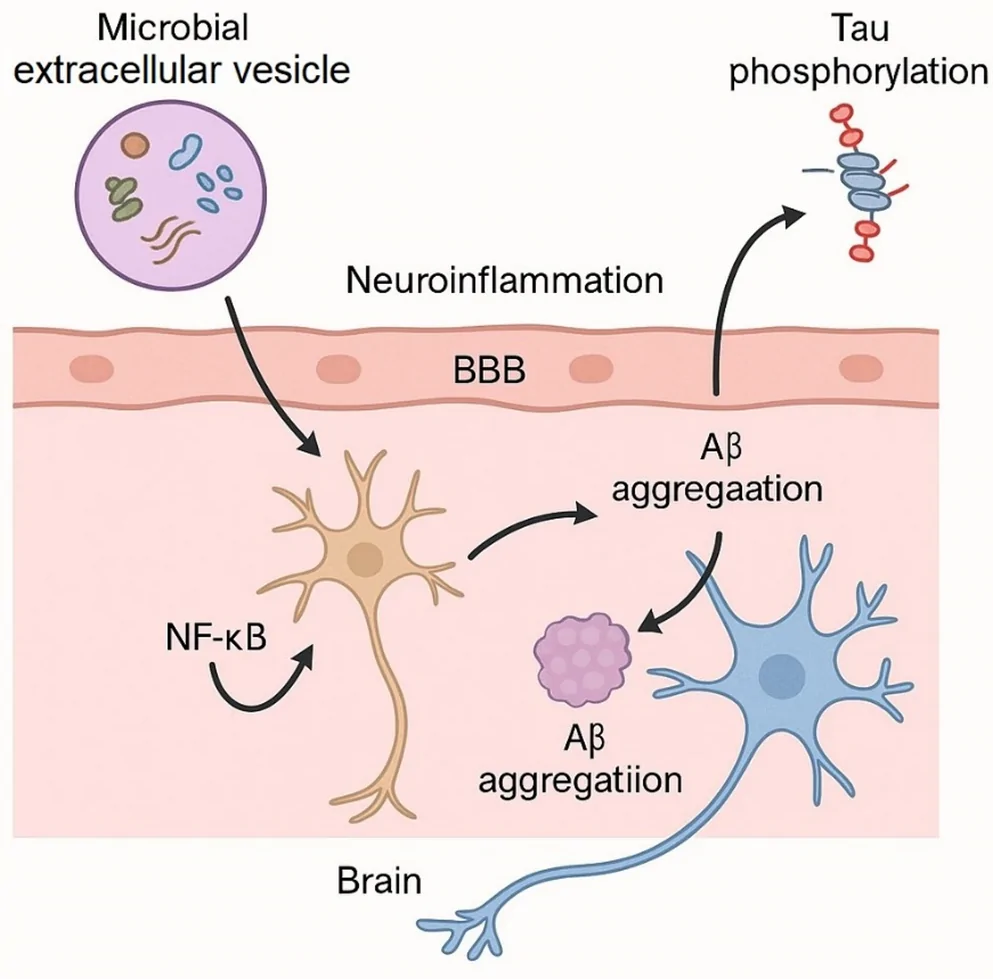
Molecular pathways of microbial extracellular vesicle (EV)-induced Alzheimer’s pathology. Microbial EVs derived from periodontal pathogens can cross the blood–brain barrier and interact with neural cells. Within the brain, they activate NF-κB signaling in microglia, promoting neuroinflammation and cytokine release. EVs cargos contribute to amyloid-beta (Aβ) aggregation, tau hyperphosphorylation, oxidative stress, and neuronal apoptosis, ultimately leading to synaptic dysfunction and cognitive decline

The periodontal-brain axis involves complex communication mediated by both microbial EVs and host exosomes (Ivana Shawkatova et al. 2025a; Sun and Chen 2024). Oral pathogens like *P. gingivalis* primarily release OMVs that transfer virulence factors, such as gingipains, from the periphery into the CNS (Fig. 3) (Ivana Shawkatova et al. 2025a). Studies suggest that microbial-induced host exosomes, originating from *P. gingivalis*-infected cells, can also carry virulence factors, crossing the BBB and inducing inflammatory responses (Ivana Shawkatova et al. 2025a). Once in the brain, these microbial cargo carriers interact with host glial cells (Butler et al. 2024; Ivana Shawkatova et al. 2025a). Simultaneously, host exosomes derived from neurons, astrocytes, or microglia participate in AD by either spreading pathogenic proteins or promoting Aβ clearance (Sun and Chen 2024; Zhao et al. 2023). Viewed together, microbial vesicles (*A*. *actinomycetemcomitans* OMVs) deliver RNA that activates NF-κB (Ha et al. 2020; Han et al. 2019), while host exosomes from mesenchymal stem cells (MSCs), astrocytes, and neural stem cells supply miRNAs and proteins that restrain inflammation, apoptosis, and mitochondrial decline (Du et al. 2021; Duan et al. 2020; B. Li et al. 2024; H. Wei et al. 2020a, b). This reciprocal interplay suggests that AD pathogenesis may depend on the balance between pathogen-driven EVs insults and host exosomal neuroprotection, offering converging therapeutic targets aimed at shifting the vesicle milieu toward repair rather than degeneration.

## Current Controversies and Conflicting Findings

The literature reveals several unresolved controversies. Some studies report that bacterial EVs or host-derived exosomes increase Aβ aggregation and tau phosphorylation, while others show minimal or no neurotoxic effects. Conflicting results also exist regarding exosomes’ capacity to cross the BBB, with discrepancies likely attributable to differences in vesicle size, surface charge, and delivery route. Additionally, while several authors interpret exosomal miRNAs as causal mediators of AD pathology, most evidence remains associative. Moreover, there is contradict across studies in terms of the relative contribution of bacterial vs. host-derived vesicles in the AD pathology. These contradictions underscore the need for harmonized methodologies and caution against overinterpreting preliminary mechanistic findings.

### Clinical Implications and Future Directions

Translational evidence strongly links oral microbial dysbiosis, particularly involving *Porphyromonas* species, to the pathogenesis of AD and cognitive impairment (Adnan et al. 2025; Chaple-Gil et al. 2025). Preclinical models demonstrate that oral microbe-induced EVs and OMVs cross the BBB and deliver pathogenic cargo, such as gingipains, initiating neuroinflammation and cognitive deficits (Butler et al. 2024; Elashiry et al. 2024; Wu et al. 2025a, b). Clinically, *P. gingivalis* components are consistently detected in AD patient brains (Chaple-Gil et al. 2025; Wu et al. 2025a, b). Furthermore, host-derived exosomes serve dual purposes: as diagnostic biomarkers (liquid biopsy) for preclinical AD (pAD) through cargo profiling (Aβ, tau, miRNAs) (Liu and Geng 2024; Titze-de-Almeida et al. 2025; Wang et al. 2022), and as therapeutic agents, notably MSC secretome, which has demonstrated safety and improved cognitive scores in human clinical trials (Liu and Geng 2024; Morita et al. 2024; Ruan et al. 2020).

#### In Vitro Evidence

In vitro studies demonstrate that oral pathogen EVs directly compromise the neurovascular unit. *P. gingivalis* OMVs carry gingipains which are internalized by human brain microvascular endothelial cells (BMECs) (Wu et al. 2025a, b). This uptake leads to the degradation of key intracellular tight junction proteins, specifically ZO-1 and occludin, thereby disrupting BBB integrity and decreasing electrical resistance (Butler et al. 2024; Wu et al. 2025a, b). Human PD-derived EVs (PD EVs) containing pro-inflammatory cytokines such as IL-1β and IL-6 also promoted BBB permeability in a 3D model (Elashiry et al. 2024). Moreover, *P. gingivalis* OMVs trigger the activation of the NLRP3 inflammasome and induce cytokine production in microglia-like cells, confirming their potential to drive neuroinflammation (Butler et al. 2024). Also, human cell–based models show that microbe-derived secretions — including metabolites and vesicles — directly alter neuronal maturation and inflammatory signalling. Kim et al. (N. Y. Kim et al. 2024) used a gut–brain chip with iPSC-derived neurons to show strain-specific microbial metabolites and EVs modulate NeuroD1 expression, synaptogenesis (GAP43/PSD95) and neuronal maturation, supporting a direct trophic or maladaptive effect of microbial products on neural cells. Kandpal et al. (2024) complement this by showing *H. pylori* secretome activates STAT3 and elevates APP/APOE4 and ROS in neuronal and neuron–astrocyte co-cultures, linking bacterial secretions to pro-amyloid and pro-inflammatory programmes. Together these in vitro data define mechanistic entry points (synaptic plasticity, STAT3, oxidative stress) that justify in vivo investigation.

### Animal Models

Animal models provide robust evidence for the translocation and neurotoxicity of oral EVs. Intragingivally injected PD EVs from *P. gingivalis*-infected mice successfully crossed the BBB and localized to microglial cells in the hippocampus of recipient uninfected mice (Elashiry et al. 2024). These PD EVs delivered *P. gingivalis* virulence factors, including Rgp and Mfa-1, to the brain (Elashiry et al. 2024). Furthermore, oral gavage of *P. gingivalis* OMVs administered to middle-aged mice induced memory dysfunction, neuroinflammation, and enhanced tau protein phosphorylation in the cortex and hippocampus (Butler et al. 2024; Wu et al. 2025a, b). Therapeutically, neural stem cell-derived EVs (NSC-EVs) rescued cognitive deficits in 9-month-old APP/PS1 mice by enhancing mitochondrial function and increasing Sirtuin 1 (SIRT1) levels, critically without altering Aβ concentrations (Li et al. 2020; Phelps et al. 2025). Preclinical studies provide causal evidence that oral microbes and exosome-like vesicles perturb brain barriers and accelerate AD-like pathology. Jiang et al. (2025) report that topical oral *P. gingivalis* induces PD with increased BBB permeability, impaired meningeal lymphatic drainage (reduced LYVE1), immune cell and bacterial brain infiltration, tau hyperphosphorylation (via reduced p-Akt/p-GSK3β) and neuronal loss—linking barrier dysfunction to amyloid/tau pathways. NSC- or stem cell–derived EV therapies also show benefit: Li et al. (2020) (and related 2024 stem-cell exosome work) improved cognition (*p* < 0.01), boosted SIRT1/PGC-1α–driven mitochondrial biogenesis and synaptic markers (*p* < 0.01) in APP/PS1 mice without reducing Aβ, indicating functional rescue through metabolic and synaptic support rather than amyloid clearance. Yet, a large part of these results originate from animal research, and it remains unclear how applicable these mechanisms are to human AD.

#### Human Observational Studies

Human observational studies consistently link oral dysbiosis to cognitive impairment (Table 3) (Adnan et al. 2025; Chaple-Gil et al. 2025). Specific periodontal pathogens such as *T. forsythia*, *F. nucleatum*, and *Porphyromonas* species are frequently associated with AD and mild cognitive impairment (MCI) (Chaple-Gil et al. 2025). Crucially, direct evidence shows *P. gingivalis* DNA and its gingipain virulence factors (RgpB and Kgp) localized within the cortical gray matter and basal forebrain of post-mortem AD patient brains (Chaple-Gil et al. 2025; Liu et al. 2024; Wu et al. 2025a, b). Reduced overall microbial diversity is also reported in individuals with AD (Chaple-Gil et al. 2025). Translational findings indicate that distinct salivary microbiome and proteome signatures can differentiate between stages of cognitive decline, highlighting their potential as non-invasive biomarkers (Adnan et al. 2025; Chaple-Gil et al. 2025; Wang et al. 2022). Additionally, oral health interventions like chlorhexidine gluconate have demonstrated the ability to alter oral flora composition (Cao et al. 2025). Large cohort and biomarker studies yield associative but clinically relevant signals. Adnan et al. (2025) found oral-niche specific microbiome differences (lower *Gemella*, higher anaerobic pro-inflammatory taxa) correlate with worse cognitive Z-scores in older adults, suggesting clinically measurable oral dysbiosis-cognition links. Abner et al. (2020) showed endothelial-derived plasma exosomes (EDEs) are enriched for Aβ40/42, p-tau and PrPᶜ in preclinical/MCI subjects with small-vessel disease, supporting EDE cargo as early vascular-AD markers.

**Table 3:** Preclinical and clinical evidence of extracellular vesicle involvement in AD

Study type	Sample size/model	Extracellular vesicle (EV) findings	Cognitive/neurological outcomes
Animal model (pathogenesis/translocation)	C57B6 mice (6 months), intragingival injection model (Elashiry et al. 2024).	PD-Derived EVs (PD EVs) carrying *P. gingivalis* virulence factors (RGP, Mfa-1) crossed the BBB and localized to hippocampal microglial cells (Elashiry et al. 2024).	Demonstrated vehicle for dissemination of oral microbial virulence factors to the CNS (Elashiry et al. 2024).
Animal model (pathogenesis/neurotoxicity)	Middle-aged mice.	*P. gingivalis* OMVs localized to the hippocampus and cortex (Butler et al. 2024; Wu et al. 2025a, b). Activated NLRP3 inflammasome (Butler et al. 2024).	Induced memory dysfunction, neuroinflammation, and tau protein phosphorylation (Butler et al. 2024; Wu et al. 2025a, b).
Animal model (therapeutic)	9-month-old APP/PS1 mice.	Neural Stem Cell-derived EVs enhanced mitochondrial function (PGC1α, NRF1, Fis1), increased SIRT1 levels, and decreased inflammatory/oxidative markers (Iba1, 4-HNE) (Li et al. 2020; Phelps et al. 2025). Aβ level was not altered (Li et al. 2020; Phelps et al. 2025).	Exhibited significant improvement in cognitive performance and improved synaptic morphology (Li et al. 2020; Phelps et al. 2025).
Animal model (therapeutic/inhibition)	Tauopathy transgenic mouse model (P301S mice) (Asai et al. 2015; Sarkar et al. 2016).	Inhibition of exosome synthesis (using GW4869) dramatically suppressed tau propagation (Asai et al. 2015; S. Chen et al. 2023a; Ding et al. 2022; Sarkar et al. 2016).	Reduced excitability in the dentate gyrus and halted the spread of tau pathology (Asai et al. 2015; Sarkar et al. 2016).
Animal model (therapeutic/engineered)	AD mice	Fe65-engineered neuronal exosomes encapsulating corynoxine-B were administered (Liu and Geng 2024; Sadeghi et al. 2025). Glycosphingolipid-enriched exosomes reduced Aβ deposition (Daksh et al. 2025; Yuyama et al. 2014).	Ameliorated cognition and pathology of AD (Liu and Geng 2024; Sadeghi et al. 2025). Enhanced microglial clearance (Yuyama et al. 2014).
Human case-control/cohort (diagnosis)	AD (*n* = 57), FTD (*n* = 16), and longitudinal cohort (*n* = 24) (Abner et al. 2020; Daksh et al. 2025; Fiandaca et al. 2015; Ghosh et al. 2025; Titze-de-Almeida et al. 2025; Wang et al. 2022).	Neurally derived blood exosomes profiled pathogenic proteins: Aβ, T-tau, and P-T181-tau (Daksh et al. 2025; Fiandaca et al. 2015; Liu and Geng 2024; Titze-de-Almeida et al. 2025). Elevated BACE-1 found in astrocyte-derived exosomes (ADEs) (Goetzl et al. 2016; Hassan et al. 2024).	Identified profiles capable of recognizing preclinical AD (pAD) (Abner et al. 2020; Daksh et al. 2025; Fiandaca et al. 2015; Ghosh et al. 2025; Liu and Geng 2024; Wang et al. 2022).
Human clinical trial (therapy)	Patients with mild-to-moderate AD (*n* = 13) (Liu and Geng 2024; Morita et al. 2024).	Intranasal administration of adipose-derived MSC secretome (including exosomes/HGF) (Liu and Geng 2024; Morita et al. 2024; Ruan et al. 2020).	Showed significant improvement in cognitive performance (HDS-R scores improved from 15.6 ± 1.0 to 17.5 ± 1.4) (Morita et al. 2024; Ruan et al. 2020).
Human observational (diagnosis/CSF)	CSF samples from controls (*n* = 9), mild AD (*n* = 10), moderate AD (*n* = 7) (Fiandaca et al. 2015; Saman et al. 2012).	Percentage of phosphorylated tau (P-T181-tau) showed a marked spike specifically in the exosomal fraction of CSF in early AD (Braak stage 3) (Fiandaca et al. 2015; Saman et al. 2012).	Differentiated early clinical stages of AD (MCI/Braak Stage 3) from controls and later stages (Fiandaca et al. 2015; Saman et al. 2012).

AD: Alzheimer’s disease; OMV: outer membrane vesicle; PD: periodontitis; ADEs: astrocyte-derived exosomes; MSC: mesenchymal stem cell; CSF: cerebrospinal fluid

#### Extracellular Vesicles Biomarkers

Exosomes are highly valuable for liquid biopsy because neurally derived exosomes (NDEs) bypass the BBB, carrying pathogenic cargo that mirrors the CNS environment (Daksh et al. 2025; Gámez-Valero et al. 2019; Liu and Geng 2024). The assessment of pathogenic proteins within NDEs isolated from blood plasma aids in identifying pAD (Abner et al. 2020; Daksh et al. 2025; Fiandaca et al. 2015; Ghosh et al. 2025; Liu and Geng 2024; Titze-de-Almeida et al. 2025; Wang et al. 2022). Key biomarkers include quantified levels of Aβ, total tau, and phosphorylated tau (P-T181-tau) (Daksh et al. 2025; Fiandaca et al. 2015; Liu and Geng 2024; Titze-de-Almeida et al. 2025). Elevated levels of β-secretase (BACE-1) were detected specifically in ADEs in AD patients compared to controls (Goetzl et al. 2016; Hassan et al. 2024). Furthermore, CSF analysis revealed a marked spike in the proportion of P-T181-tau in the exosomal fraction during the earliest clinical stages of AD (Braak stage 3/MCI) (Fiandaca et al. 2015; Liu and Geng 2024; Saman et al. 2012; Titze-de-Almeida et al. 2025). Altered exosomal microRNA (miRNA) profiles, notably reduced serum exosomal miR-185-5p, also serve as differentiating biomarkers (Daksh et al. 2025; Ding et al. 2022; Liu and Geng 2024). Also, EDE enrichment for amyloid and p-tau (Abner et al. 2020) and niche-specific oral microbiome signatures (Adnan et al. 2025) indicate combined vesicle/microbiome panels could enhance early detection—particularly for vascular-contributing AD phenotypes.

#### Therapeutic Potential of Extracellular Vesicles

Exosomes act as both pathological vectors and therapeutic delivery platforms. Delivery platforms can improve the therapeutic effects of compounds (Alavi et al. 2024a, b, c; Alavi et al. 2019, 2020, 2021, 2022a, b, 2024a, b, c; Ebrahimi Shahmabadi et al. 2014; Ghaferi et al. 2024). They mediate the cell-to-cell spread of aggregated proteins like tau and α-synuclein (prionoid propagation) (Asai et al. 2015; Daksh et al. 2025; Ding et al. 2022; Wang et al. 2017). Targeting this process, pharmacological inhibition of exosome synthesis using GW4869 successfully halted tau propagation in vivo and was associated with lower amyloid plaque load in mouse AD models (Asai et al. 2015; Daksh et al. 2025; Ding et al. 2022; Dinkins et al. 2014; Sarkar et al. 2016; Titze-de-Almeida et al. 2025). For therapeutic delivery, MSC-derived exosomes offer neuroprotective potential (Phelps et al. 2025; Sadeghi et al. 2025). In human clinical trials, the non-invasive intranasal administration of adipose-derived MSC secretome (including exosomes) led to a significant improvement in cognitive performance (HDS-R scores) in patients with mild-to-moderate AD (Liu and Geng 2024; Morita et al. 2024; Ruan et al. 2020). Additionally, engineered neuronal exosomes, such as Fe65-engineered vesicles carrying corynoxine-B, improved cognition and pathology in AD mice (Liu and Geng 2024; Sadeghi et al. 2025). Proof-of-principle exists for both engineered delivery and cell-derived therapeutics: Alvarez-Erviti et al. (Alvarez-Erviti et al. 2011) demonstrated RVG-targeted exosomes deliver siRNA and knock down BACE1 in brain, and a phase I/II intranasal allogeneic MSC-exosome trial (X. Xie et al. 2023a) reported safety and suggestive cognitive signal. Preclinical NSC/MSC-EV studies (Li et al. 2020) show mechanistic rescue via mitochondrial, anti-inflammatory and anti-apoptotic pathways. Altogether, translational evidence is promising but heterogeneous—human causality, optimal dosing, targeting strategies, and long-term outcomes remain to be established.

### Critical Appraisal of the Evidence

The study of exosomal mechanisms in the periodontal-brain axis offers substantial translational potential, particularly for minimally invasive diagnostics (Bolívar et al. 2024; Ghosh et al. 2025; Titze-de-Almeida et al. 2025). A major strength is the capability of utilizing exosomal cargo (mRNAs, ncRNAs) combined with machine learning (ML) to accurately predict AD diagnosis and age of onset (Bolívar et al. 2024; Mosquera-Heredia et al. 2024). However, key challenges persist, notably the pervasive methodological heterogeneity in EV research due to the lack of standardized isolation protocols (Da Conceicao et al. 2025; Ghosh et al. 2025; Louka and Koumandou 2024). While compelling preclinical evidence suggests that oral microbial OMVs and their virulence factors cross the BBB and induce pathology (Liu et al. 2024; Wu et al. 2025a, b), the field is continuously struggling to definitively establish causality versus correlation between peripheral oral infection and central neurodegeneration (Hambarsari et al. 2025; Liu et al. 2024; Wu et al. 2025a, b).

#### Strengths and Novelty of Current Findings

The primary strength of studying EVs is their natural capacity to transport molecular signatures across the BBB, providing a non-invasive window into the CNS pathological state via peripheral fluids (liquid biopsy potential) (Da Conceicao et al. 2025; Ghosh et al. 2025; Mosquera-Heredia et al. 2024). This minimally invasive approach facilitates the diagnosis and monitoring of AD (Daksh et al. 2025; Titze-de-Almeida et al. 2025; Uceda et al. 2025). Furthermore, research has achieved high novelty using ML to analyze exosomal mRNA expression profiles (e.g., GABRB3, CADM1, TNFRSF19), yielding diagnostic accuracy exceeding 90% for AD and correlating key transcripts (LIMK2) with the age of onset (ADAOO) (Bolívar et al. 2024; Mosquera-Heredia et al. 2024). Specifically addressing the oral axis, the demonstrated mechanism by which microbial OMVs carry gingipains to the brain, activate neuroinflammation, and promote tau phosphorylation in animal models establishes a vital, mechanistic link (Liu et al. 2024; Wu et al. 2025a, b).

Collectively, these studies provide mechanistic and translational insight into how microbial EVs and host exosomes contribute to AD pathology. Gong et al. (2022) suggested that oral delivery of *P. gingivalis* OMVs impaired learning and memory, reduced tight-junction proteins (ZO-1, occludin, claudin-5), and triggered NLRP3-dependent tau phosphorylation in mice—directly linking a periodontal pathogen to hallmark AD lesions. Palacios et al. (2023) extended the concept to *H. pylori*, showing that purified OMVs cross epithelial barriers, accumulate in brain tissue, and drive NF-κB–dependent astrocyte reactivity and neuronal damage. On the human side, Manolopoulos et al. (2023) found that neuron-derived EVs (NDEVs) carrying Aβ42/Aβ40 and proBDNF accurately distinguished MCI-DEM and DEM-DEM from NRM-NRM groups, while Bei et al. (2023) revealed that circulating AD exosomes disrupt VE-cadherin and weaken the BBB. These data collectively highlight exosomes as both effectors and biomarkers of AD pathogenesis. Nevertheless, a significant portion of these findings are derived from animal studies, and it is still uncertain how relevant these mechanisms are to human AD.

#### Methodological Challenges (Isolation, Reproducibility)

A major impediment to clinical translation is the fragmentation of methodologies and the inherent complexity of EVs (Ghosh et al. 2025; Uceda et al. 2025). Current technologies suffer from limitations in reliably distinguishing between EV subtypes (exosomes, microvesicles) (Louka and Koumandou 2024; Welsh et al. 2024). This ambiguity is compounded by the lack of standardized and validated isolation protocols, which hinders reproducibility and inter-laboratory comparison (Da Conceicao et al. 2025; Ghosh et al. 2025; Hambarsari et al. 2025; Titze-de-Almeida et al. 2025). Isolation of low-abundance brain-derived EVs (NDEVs) in peripheral fluids remains challenging due to the difficulty in identifying standardized, validated immunocapture targets (Da Conceicao et al. 2025; Wu et al. 2025a, b). Additionally, adherence to standardized reporting guidelines, such as MISEV (Minimal Information for Studies of Extracellular Vesicles), is necessary to improve reproducibility (Mosquera-Heredia et al. 2024; Phelps et al. 2025). Finally, cell culture studies must mitigate contamination from bovine EVs present in standard serum supplements (Phelps et al. 2025). Isolation approaches vary widely—oral gavage versus systemic injection in animals, size-exclusion or immuno-capture for human plasma—introducing heterogeneity in vesicle yield, purity, and RNA/protein cargo. Small sample sizes (e.g., Bei et al. (2023) *n* = 5 per group) and limited replication raise concerns about reproducibility and effect-size inflation. Throughout this review, we synthesize methodological strengths, weaknesses, and recurring experimental limitations from the literature. Differences in EV isolation, purification, and characterization significantly affect findings, and these details were systematically extracted and compared to ensure an accurate and critical synthesis. Across published studies (Kowal et al. 2016; Ljungström and Oltra 2025; Théry et al. 2018; Tian et al. 2020), methodological variability significantly affects the interpretation of findings. For example, bacterial EVs have been isolated using ultracentrifugation, size-exclusion chromatography, or polymer-based precipitation, each yielding different vesicle purity and size distributions. Proteomic and miRNA studies rely on diverse platforms—including NGS, qPCR panels, mass spectrometry, and nanoparticle tracking analysis—leading to heterogeneity in reported exosomal cargo (Kowal et al. 2016; Ljungström and Oltra 2025; Théry et al. 2018; Tian et al. 2020). Animal models also vary widely, ranging from intranasal EV administration to systemic injection and genetically predisposed AD mice models (Arjmand et al. 2026; Ma et al. 2023). These methodological disparities limit cross-study comparability and may partially explain conflicting conclusions regarding EV neurotoxicity and permeability across the BBB (Arjmand et al. 2026; Kowal et al. 2016; Ljungström and Oltra 2025; Ma et al. 2023; Théry et al. 2018; Tian et al. 2020; Tkach and Théry 2016; Willms et al. 2018). Interpretation of these findings should also consider the methodological variability in EV isolation and characterization across studies, which may influence the reported composition and biological effects of vesicle populations.

#### Conflicting or Inconclusive Evidence

The functional role of EVs remains complex, often described as a “double-edged sword,” mediating both neuroprotection and the spread of pathogenic proteins like Aβ and tau (Da Conceicao et al. 2025; Daksh et al. 2025; Soleymani et al. 2023; Titze-de-Almeida et al. 2025; Uceda et al. 2025). This dual nature can lead to inconclusive functional interpretations. In clinical translation, results remain mixed; while certain stem cell secretome-based therapies (containing exosomes) led to significant improvements in cognitive scores (ADAS-cog, MoCA-B) in AD patients (Hambarsari et al. 2025; Phelps et al. 2025), other clinical trials reported adverse effects, albeit transient, such as fever (43%) and headaches (33%) (Hambarsari et al. 2025). Furthermore, a fundamental issue in the oral axis is that while *P. gingivalis* nucleic acids and virulence factors (gingipains) are consistently detected in AD patient brains (Liu et al. 2024; Wu et al. 2025a, b), attempts to recover viable, whole oral bacteria from human brain tissue have failed, making the source and progression of the infection unclear (Liu et al. 2024; Wu et al. 2025a, b). Although *P. gingivalis* OMVs and *H. pylori* OMVs both activate glia and damage BBB integrity, their downstream pathways differ (NLRP3 inflammasome versus NF-κB). Manolopoulos et al. (2023) observed declining Aβ42/Aβ40 ratios in both plasma and NDEVs, but correlations between the two biofluids were absent, highlighting variability across compartments. Although these observations support a potential link between periodontal pathogens and neuroinflammation, the relatively small sample sizes and cross-sectional design of many human studies limit the ability to infer causality.

#### Causality Vs. Correlation

Establishing causality between oral microbial EVs and AD pathogenesis is the critical remaining challenge, as much of the current evidence relies on strong correlation (Hambarsari et al. 2025; Wu et al. 2025a, b). The current mechanistic evidence supports the concept of *P. gingivalis* acting as a focal infection, where its OMVs carrying components like gingipains are disseminated systemically (Liu et al. 2024). Once crossing the BBB, these OMVs induce neuroinflammation, accelerate tau phosphorylation, and are implicated in disrupting iron homeostasis, thereby promoting AD-like pathology (Liu et al. 2024; Wu et al. 2025a, b). However, host-derived exosomes contribute to established pathology by facilitating the prion-like propagation and intercellular transfer of already misfolded Aβ and tau proteins throughout the CNS (Da Conceicao et al. 2025; Daksh et al. 2025; Ghosh et al. 2025; Mosquera-Heredia et al. 2024; Titze-de-Almeida et al. 2025; Uceda et al. 2025). Longitudinal clinical validation is imperative to determine if microbial EVs are initiators or powerful accelerators of pre-existing neurodegeneration (Hambarsari et al. 2025). Animal models provide causal links between microbial vesicles and AD-like pathology, but human studies remain largely associative. For instance, Manolopoulos et al. (2023) in a nested case-control study, suggested that reduced NDEV proBDNF or plasma Aβ ratios could be consequences rather than drivers of neurodegeneration (Manolopoulos et al. 2023).

#### Comparison with Established Alzheimer’s Risk Factors

Established AD risk factors are dominated by aging and genetic predisposition, primarily the presence of the *APOE4* allele (Bolívar et al. 2024; Liu et al. 2024; Titze-de-Almeida et al. 2025). These factors contribute to the accumulation of classical AD hallmarks (Aβ and tau) (Bolívar et al. 2024; Titze-de-Almeida et al. 2025). The oral EV hypothesis integrates systemic chronic inflammation and microbial dysbiosis as potentially modifiable environmental risk factors that interface with these core pathways (Bolívar et al. 2024; Titze-de-Almeida et al. 2025; Wu et al. 2025a, b). EVs biomarkers significantly advance the conventional biological definition of AD (ATN framework) by providing reliable quantification of canonical markers like Aβ42, total tau, and phosphorylated tau (P-T181-tau) in easily accessible blood samples (Daksh et al. 2025; Soleymani et al. 2023; Titze-de-Almeida et al. 2025). Furthermore, novel EV signatures, particularly mRNA profiles identified using ML, can provide predictive risk for AD onset, surpassing traditional reliance solely on genetics and age-related decline (Bolívar et al. 2024; Mosquera-Heredia et al. 2024). While APOE ε4, age, and vascular comorbidities remain dominant predictors of AD (Bolívar et al. 2024; Liu et al. 2024; Titze-de-Almeida et al. 2025), these EVs and exosome studies (Bei et al. 2023; Manolopoulos et al. 2023) suggest complementary pathways—periodontal infection and exosome/EVs-mediated BBB dysfunction—that may amplify classical risks and offer novel diagnostic or therapeutic targets beyond traditional genetic and vascular factors.

### Future Directions and Research Needs

Future research must prioritize validating EVs/exosomal mRNA and ncRNA biomarkers in larger, diverse cohorts to confirm reliability and clinical utility (Bolívar et al. 2024; Mosquera-Heredia et al. 2024; Youssef et al. 2025). Overcoming methodological barriers, particularly the standardization of isolation and characterization protocols (MISEV), is critical for clinical translation (Ghosh et al. 2025; Jia et al. 2022; Uceda et al. 2025; Welsh et al. 2024; Youssef et al. 2025). Advanced integration of artificial intelligence (AI) and multi-omics analysis is crucial to understanding complex molecular mechanisms and optimizing therapeutic strategies, including periodontal intervention trials (Hambarsari et al. 2025; Lu et al. 2025; Lundergan et al. 2024; Uceda et al. 2025).

#### Standardization of Extracellular Vesicle Methodologies

The primary hurdle is the lack of standardized protocols for isolating and characterizing EVs (Ghosh et al. 2025; Gorgzadeh et al. 2024; Jia et al. 2022; Lu et al. 2025; Titze-de-Almeida et al. 2025; Youssef et al. 2025). This compromises purity, yield, and reproducibility across studies (Gorgzadeh et al. 2024; Jia et al. 2022; Li et al. 2025; Titze-de-Almeida et al. 2025; Welsh et al. 2024). Future efforts must focus on adhering to the MISEV guidelines (Minimal Information for Studies of Extracellular Vesicles) to establish rigorous nomenclature and purity criteria (Ghosh et al. 2025; Welsh et al. 2024; Youssef et al. 2025). Standardization should involve utilizing orthogonal detection methods to achieve traceable concentration measurements and developing Good Manufacturing Practice (GMP)-compliant production pipelines for scalable clinical use (Lai et al. 2024; Lu et al. 2025; Welsh et al. 2024; Youssef et al. 2025). Comparative isolation studies expose large method-dependent variability that undermines reproducibility. Sou et al. (2025) directly compared differential ultracentrifugation (DUC), polyethylene glycol (PEG)-based precipitation, and a combination of both (PEG + UC) on human serum and found PEG + UC delivered the best trade-off of yield and purity while preserving miRNA recovery, whereas DUC gave low yield/purity and PEG high yield but low purity. Bharti et al. (2025) propose a cocktail + two-step filtration workflow producing highly homogeneous small EVs (sEVs) compatible with downstream multi-omics. Together these papers argue strongly for adopting harmonized, benchmarked pipelines (e.g., PEG + UC or validated cocktail protocols) and for mandatory orthogonal characterization (particle: protein, marker panels, RNA recovery) in AD EV studies.

#### Longitudinal Human Studies and Intervention Trials

Larger-scale longitudinal studies are critical to validate identified exosomal/EVs mRNA and ncRNA biomarkers across diverse populations and confirm their robustness in clinical settings (Bolívar et al. 2024; Mosquera-Heredia et al. 2024; Youssef et al. 2025). Future efforts must include randomized controlled clinical trials (RCTs) to establish definitive causation and evaluate if periodontal interventions (e.g., gum treatment or oral health programs) significantly alter AD progression or cognitive outcomes (Lundergan et al. 2024; Qi et al. 2025). Such trials should use the accepted case definition for PD and control for confounding variables (Lundergan et al. 2024). Furthermore, assessing the value of periodontal biomarkers (e.g., pathogen IgG antibodies) for predicting AD risk is needed (Lundergan et al. 2024). Delgado-Peraza et al. (Delgado-Peraza et al. 2023) provide a model for intervention-embedded plasma NDEVs research: exercise increased neuroprotective cargo (proBDNF, BDNF, humanin) in plasma NDEVs (*p* < 0.05) and showed APOE-dependent responsiveness. These results demonstrate feasibility of serial NDEV measures in RCTs and underscore the need for longer follow-up, larger samples, and stratification by genetic risk to test causality and treatment responsiveness.

#### Integration of Multi-Omics Approaches

Integrating multi-omics approaches (transcriptomics, proteomics, metabolomics) is essential for developing a systems-level disease model and improving biomarker discovery (Gézsi et al. 2019; Lu et al. 2025; Youssef et al. 2025). Analyzing multi-omics data from EVs helps elucidate complex disease mechanisms, particularly identifying intricate miRNA-mRNA pathways and circRNA integrations involved in AD pathophysiology (Besli et al. 2023; Lu et al. 2025). Future studies require specialized computational modeling to handle the high-throughput and heterogeneous nature of omics data (Lu et al. 2025; Picchio et al. 2025). This comprehensive strategy contributes to a better understanding of the complex interplay between peripheral microbial signals and central neurodegenerative processes (Uceda et al. 2025). Rai et al. (2024) mapped plasma EV proteomes (≈ 4,500 proteins) and lipidomes (829 lipids), identifying conserved EV markers (ADAM10, PS(36:1) and separating EV from non-EV particles—an exemplar for deep, quantitative EV atlases. Cohn et al. (2021) showed cell-type (microglial) EV multi-omics can reveal disease-specific lipid, protein and miRNA signatures (e.g., increased tau, FTH1, TREM2; altered DHA-containing lipids). These studies argue for routine integration of proteomics, lipidomics and small-RNA sequencing on the same well-characterized vesicle fractions.

#### Artificial Intelligence and Systems Biology for Extracellular Vesicle Research

The convergence of AI and EV research is transformative, providing new tools for data integration and pattern analysis (Lu et al. 2025; Picchio et al. 2025; Uceda et al. 2025). AI, specifically ML, is crucial for analyzing multi-omics data, unveiling correlations between EV cargo and neuroinflammatory progression, and achieving highly sensitive biomarker identification (Ghosh et al. 2025; Lu et al. 2025; Picchio et al. 2025; Uceda et al. 2025; Youssef et al. 2025). AI is necessary to expedite the design and optimization of engineered therapeutic EVs by automating experimental design and simulating organismal behaviors in the design-build-test-learn cycle (Lu et al. 2025). Future research should utilize AI to establish highly accurate prediction models for AD risk and progression (Bolívar et al. 2024; Xie et al. 2025). ML and systems biology are increasingly applied to decipher complex EV datasets. Arora and Raghava (2025) used ensemble AI models to predict exosomal miRNAs with an AUC of 0.73 and developed the EmiRPred web server for motif discovery. Li et al. (Y. Li et al. 2024) achieved 94.4% accuracy distinguishing exosomes from six cell lines by combining SERS spectra with PCA-SVM. Such approaches promise automated classification of exosomes by origin or disease state and integration of multi-omic layers into predictive disease networks.

#### Translational Potential: Periodontal Therapy in AD Prevention

**Fig. 4:**
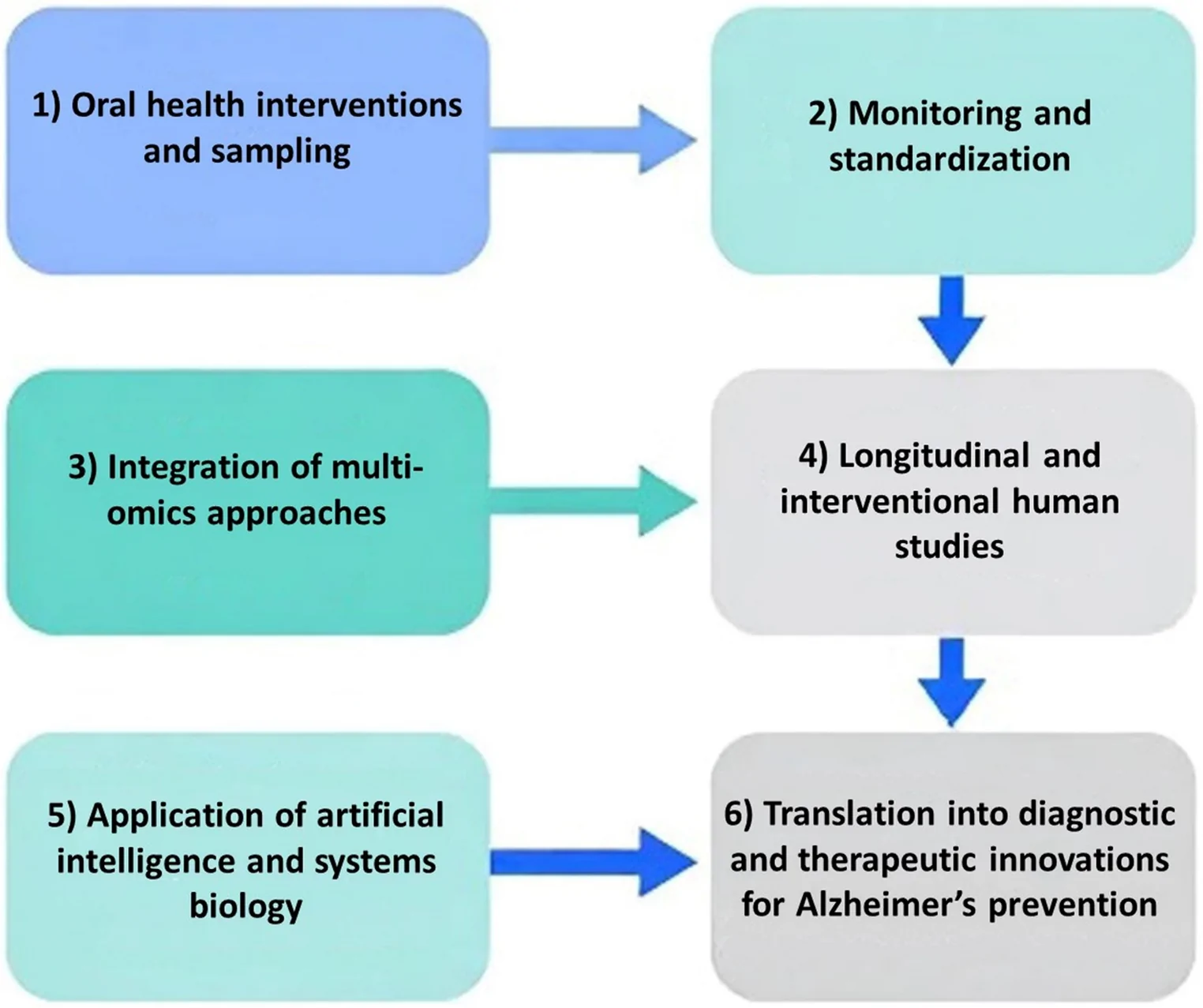
Future research roadmap for microbial extracellular vesicles in Alzheimer’s disease. Proposed trajectory for advancing research on the periodontal–brain axis. Key stages include oral health interventions and sampling, monitoring and standardization, integration of multi-omics approaches, longitudinal and interventional human studies, application of artificial intelligence and systems biology, and translation into diagnostic and therapeutic innovations for Alzheimer’s prevention

Given that PD is a modifiable risk factor linked to neuroinflammation, maintaining oral health is a critical prevention strategy (Inchingolo et al. 2025; Lundergan et al. 2024). Studies suggest that periodontal treatment can positively affect AD-related brain atrophy markers and slow cognitive decline in mild AD patients (Inchingolo et al. 2025; Lundergan et al. 2024). Future research must conduct RCTs to definitively confirm the clinical efficacy of periodontal therapy on AD progression (Fig. 4) (Lundergan et al. 2024; Qi et al. 2025). Furthermore, innovative therapeutic strategies should focus on developing targeted inhibitors against shared pathological mediators, such as gingipains or the NLRP3 inflammasome, for dual treatment of both diseases (Lundergan et al. 2024). Early detection and prevention are key, as intervention may be too late once symptoms are fully manifested (Lundergan et al. 2024). Proof-of-concept clinical applications are emerging: Puletic et al. (2024) reported improved periodontal clinical endpoints and reduced local cytokines after EV therapy in PD (small n), while Delgado-Peraza et al. (2023) suggests that non-pharmacologic interventions modulate NDEV cargo. Translationally, rigorous RCTs are needed to test whether periodontal treatment or EV-directed therapies alter circulating EV neurotoxic cargos and, ultimately, cognitive trajectories.

#### Limitations and Knowledge Gaps

Despite growing interest in the periodontal–brain axis, several limitations constrain current interpretations. Many studies are cross-sectional or associative in nature, limiting causal inference. Confounding factors such as aging, socioeconomic status, comorbid vascular disease, and shared inflammatory risk profiles complicate the attribution of Alzheimer’s pathology specifically to periodontal disease. In addition, variability in periodontal disease definitions, microbial profiling methods, and EV isolation techniques contributes to inconsistent findings across studies. Future longitudinal and mechanistic investigations are required to determine whether periodontal-derived EVs play a direct pathogenic role or act as modulators within a broader neuroinflammatory context. To minimize selection bias, studies were chosen to represent diverse experimental approaches and outcomes rather than selectively highlighting supportive findings. Conflicting or null results were considered where available, and causal interpretations were avoided when evidence was predominantly associative. Potential publication bias and heterogeneity across study designs were acknowledged throughout the review.

## Conclusion

Growing evidence supports a pivotal role for oral microbial EVs in bridging chronic periodontal infection and AD pathology. PD-derived EVs—especially *P. gingivalis* OMVs—carry virulence factors such as gingipains, LPS, and regulatory RNAs that can traverse the BBB, activate microglia, and trigger neuroinflammation, Aβ accumulation, and tau hyperphosphorylation. Host-derived exosomes add complexity, functioning both as vectors of toxic proteins and as potential neuroprotective carriers of anti-inflammatory microRNAs and enzymes. Together, these microbial and host vesicles create a dynamic communication network that amplifies neurodegeneration and may accelerate cognitive decline.

Preclinical studies consistently demonstrate that periodontal EVs reach the brain and reproduce key AD lesions, while clinical investigations reveal *P. gingivalis* DNA and gingipains in post-mortem AD tissue and distinct exosomal biomarkers in blood and CSF. Yet, significant gaps remain. Definitive proof of causality in humans, standardization of EVs/exosome isolation and characterization, and large longitudinal studies are urgently needed. The dual nature of EVs—as both pathogenic agents and therapeutic vehicles—demands careful translational strategies.

Overall, evidence suggests a plausible mechanistic connection between oral microbial EVs and AD pathology, but the strength of current findings is limited by methodological variability, small experimental sample sizes, and inconsistent analytical approaches. A unified framework—including standardized EV isolation, functional assays, and longitudinal human data—is essential before causal inferences can be confidently drawn. By critically synthesizing current knowledge and identifying contradictions and gaps, this review provides a roadmap for future research in the periodontal–brain axis. While definitive causal links remain to be established, emerging evidence supports a model in which periodontal inflammation—particularly through microbial EVs and host exosome-mediated pathways—may contribute to specific aspects of AD pathogenesis, warranting further targeted investigation. 

## Supplementary Information

Below is the link to the electronic supplementary material.


Supplementary Material 1(DOCX 16.6 KB)


## Data Availability

No datasets were generated or analysed during the current study.
