# Supplementary material for: Oral Microbial Extracellular Vesicles as Novel Mediators of Alzheimer’s Pathogenesis: A Critical Review of the Periodontal–Brain Axis
Source: Neurotox Res. 2026 Jul 16;44(4):35. doi: 10.1007/s12640-026-00802-5 (PMC13375683; doi:10.1007/s12640-026-00802-5)
Supplement: Supplementary file 1 — Supplementary Material 1(DOCX 16.6 KB) [file 12640_2026_802_MOESM1_ESM.docx]

**Supplemental Materials**

**Oral Microbial Extracellular Vesicles as Novel Mediators of Alzheimer’s Pathogenesis: A Critical Review of the Periodontal–Brain Axis**

Seyed Ebrahim Alavi^1,*^ . Hasan Ebrahimi Shahmabadi^2^ . Robert M. Love^1^ . Arun V. Kurumathur^3^ . Lavanya A. Sharma^1,*^ . Ajay Sharma^1,*^

^1^ School of Medicine and Dentistry, Griffith University, Gold Coast, Queensland, Australia

^2^ Immunology of Infectious Diseases Research Center, Research Institute of Basic Medical Sciences, Rafsanjan University of Medical Sciences, Rafsanjan, Iran

^3^ Department of Periodontics, School of Dentistry, Amrita University, Kochi, Kerala, India

**Running title:** Extracellular Vesicles in the Periodontal–Alzheimer’s Axis

*** Corresponding authors**

Seyed Ebrahim Alavi; School of Medicine and Dentistry, Griffith University, Gold Coast, Queensland, Australia; [ebrahim.alavi@griffithuni.edu.au](mailto:ebrahim.alavi@griffithuni.edu.au)

Lavanya A. Sharma; School of Medicine and Dentistry, Griffith University, Gold Coast, Queensland, Australia; [l.sharma@griffith.edu.au](mailto:l.sharma@griffith.edu.au); +61756780716

Ajay Sharma; School of Medicine and Dentistry, Griffith University, Gold Coast, Queensland, Australia; [ajay.sharma@griffith.edu.au](mailto:ajay.sharma@griffith.edu.au); +61756780733

**Table S1.** Literature search strategy used for database retrieval

| **Database** | **Search Terms** |
| --- | --- |
| PubMed | ("periodontal disease" OR periodontitis OR "oral microbiome") AND ("extracellular vesicles" OR exosomes OR "outer membrane vesicles" OR OMVs) AND ("Alzheimer's disease" OR neurodegeneration OR "blood–brain barrier" OR neuroinflammation) |
| Scopus | (periodontitis OR "oral microbiota") AND ("extracellular vesicles" OR OMVs) AND ("Alzheimer disease" OR amyloid OR tau) |
| Web of Science | ("periodontal disease" AND "extracellular vesicles") AND ("Alzheimer's disease" OR neuroinflammation) |
